# Microbial landscapes of the rhizosphere soils and roots of *Luffa cylindrica* plant associated with *Meloidogyne incognita*

**DOI:** 10.3389/fmicb.2023.1168179

**Published:** 2023-05-25

**Authors:** Ling Qu, Kang Dai, Jie Wang, Li Cao, Zhongchen Rao, Richou Han

**Affiliations:** Guangdong Key Laboratory of Animal Conservation and Resource Utilization, Guangdong Public Laboratory of Wild Animal Conservation and Utilization, Institute of Zoology, Guangdong Academy of Sciences, Guangzhou, Guangdong Province, China

**Keywords:** root-knot nematode (RKN), *Luffa cylindrica*, rhizosphere soils, plant roots *in situ*, microbial community, repellency

## Abstract

**Introduction:**

The root-knot nematodes (RKN), especially *Meloidogyne* spp., are globally emerging harmful animals for many agricultural crops.

**Methods:**

To explore microbial agents for biological control of these nematodes, the microbial communities of the rhizosphere soils and roots of sponge gourd (*Luffa cylindrica*) infected and non-infected by *M. incognita* nematodes, were investigated using culture-dependent and -independent methods.

**Results:**

Thirty-two culturable bacterial and eight fungal species, along with 10,561 bacterial and 2,427 fungal operational taxonomic units (OTUs), were identified. Nine culturable bacterial species, 955 bacterial and 701 fungal OTUs were shared in both four groups. More culturable bacterial and fungal isolates were detected from the uninfected soils and roots than from the infected soils and roots (except no fungi detected from the uninfected roots), and among all samples, nine bacterial species (*Arthrobacter* sp., *Bacillus* sp., *Burkholderia ambifaria*, Enterobacteriaceae sp., *Fictibacillus barbaricus*, *Microbacterium* sp., Micrococcaceae sp., Rhizobiaceae sp., and *Serratia* sp.) were shared, with *Arthrobacter* sp. and *Bacillus* sp. being dominant. *Pseudomonas nitroreducens* was exclusively present in the infested soils, while *Mammaliicoccus sciuri*, *Microbacterium azadirachtae*, and *Priestia* sp., together with *Mucor irregularis*, *Penicillium* sp., *P. commune*, and *Sordariomycetes* sp. were found only in the uninfected soils. *Cupriavidus metallidurans*, *Gordonia* sp., *Streptomyces viridobrunneus*, and *Terribacillus* sp. were only in the uninfected roots while *Aspergillus* sp. only in infected roots. After *M. incognita* infestation, 319 bacterial OTUs (such as *Chryseobacterium*) and 171 fungal OTUs (such as *Spizellomyces*) were increased in rhizosphere soils, while 181 bacterial OTUs (such as *Pasteuria*) and 166 fungal OTUs (such as *Exophiala*) rose their abundance in plant roots. Meanwhile, much more decreased bacterial or fungal OTUs were identified from rhizosphere soils rather than from plant roots, exhibiting the protective effects of host plant on endophytes. Among the detected bacterial isolates, *Streptomyces* sp. TR27 was discovered to exhibit nematocidal activity, and *B. amyloliquefaciens*, *Bacillus* sp. P35, and *M. azadirachtae* to show repellent potentials for the second stage *M. incognita* juveniles, which can be used to develop RKN bio-control agents.

**Discussion:**

These findings provided insights into the interactions among root-knot nematodes, host plants, and microorganisms, which will inspire explorations of novel nematicides.

## 1. Introduction

Root-knot nematode (RKN) infects the plant roots to form galls, which hinder the roots from uptaking nutrients and water and result in wilting and developmental retardation (Jones et al., 2013). RKN infection also reduces plant resistance, causes wound, favors the infection of other pathogens, and often leads to complex plant diseases (Kyndt et al., 2017; Khan and Ahamad, 2020). RKN disease has caused a large number of annual economic losses worldwide (Jones et al., 2013; Coyne et al., 2018; Khan and Ahamad, 2020).

Different methods have been employed to control these vital RKNs in integrated pest management programs, such as cultural practices, resistant crop cultivars, chemical nematicides, and biocontrol (Kepenekci et al., 2018). However, most commonly-used toxic nematicides are prohibited in field application (Abdel-Rahman et al., 2013; Maleita et al., 2017). So, novel methods for the control of harmful plant nematodes are constantly explored. Based on its environmental friendship, biological control method receives increasing attention as the most potent plant nematode control measures for maintaining the sustainable development of green agriculture (Gunasinghe et al., 2015; Kwak et al., 2018; Sun et al., 2021; Dai et al., 2022).

Rhizosphere and root-endophytic microorganisms interact with host plants, phytopathogens, and other soil occurring microorganisms (Yergaliyev et al., 2020). This interaction is fulfilled by competing nutrients, producing antimicrobial substances, and secreting volatile organic compounds (Du et al., 2022). Some of the rhizosphere and root-endophytic microorganisms are reported to affect the infection of plant pests and diseases. For example, *Bacillus firmus* YBf-10 efficiently reduces damage of *M. incognita* to tomato plants with reduced galls, egg masses on roots, and nematode counts in soil (Xiong et al., 2015). Culture supernatant of *Streptomyces hydrogenans* DH-16 lowers the root galls in the infested plants by *M. incognita* and promotes the growth of *Solanum lycopersicum* seedlings (Sharma et al., 2020). The application of *B. velezensis* BZR 86 significantly decreases the development of root-knot disease in tomato and cucumber vegetables (Migunova et al., 2021). Pot experiment suggested that *Pseudomonas simiae* strain MB751 exhibits significant nematicidal activity against *M. incognita* with approximately 80% mortality toward pre-parasitic second stage juveniles (pre-J2s; Sun et al., 2021). *Microbacterium oleivorans* triggers the innate immune responses in rice against blast disease (Sahu et al., 2021). The secondary metabolites produced by the rhizosphere and root-endophytic microorganisms also play important roles in inhibiting RKNs.

Microbial communities are analyzed from the soil associated with *Meloidogyne* spp. (Adam et al., 2014; Elhady et al., 2017), by culture-independent and -dependent approaches. The bacterial communities from nematode-infested and non-infested rhizosphere soils from four different plants (cucumber, tomato, eggplant, and bitter melon) are found to probably regulate RKN infection in plants (Zhou et al., 2019). The composition and diversity of the core microbiome associated with *M. incognita* are different according to its life stages (Cao et al., 2015).

It is known that the soil conditions or plant species influence the bacterial and fungal community (Zhou et al., 2019; Abdul Rahman et al., 2021; Sui et al., 2022). Both infested and non-infested plants by *M. incognita* have been found in the same crop locations. It is reasonable to speculate that specific microbes in non-infested and infested soil may be involved in regulating the nematode activity. As the microbes from different nematode-infected soils and plants are quite variable, in this study, microorganisms in the rhizosphere soils and roots of the sponge gourd with and without *M. incognita* infection were compared using 16S rRNA (for bacteria) and ITS (for fungi) sequences. The microbiomes of infested and non-infested rhizosphere and soils and roots were used to examine their biological potentials for the pre-J2s of *M. incognita*.

## 2. Materials and methods

### 2.1. Sample collection

All the rhizosphere soil and plant root samples of sponge gourd were collected on November 12, 2020, in Baiyun District, Guangzhou, China (113.27° N, 23.16° E). Whole plants of *L. cylindrica* were removed from the soil to observe the severity of root symptoms. Many galls were present in *M. incognita*-infested plant roots, while non-infested roots had no galls (Supplementary Figure 1). The roots were shaken gently to remove the soils that were not tightly attached, and the rhizosphere soils were collected by using a brush to separate the soils from the root system. In order to obtain endophytic bacteria, the collected roots were washed under slow-running tap water to remove adhering soil particles, followed by surface sterilization with NaClO (1%, Tianjin Best Chemical) for 5 min, successively rinsed five times with sterile distilled water, and then dried with sterile filter paper for root-endophyte isolation (Li et al., 2014). Five replicate soil and four replicate root samples from infested and non-infested plants per location were collected, with five individual samples for each replicate. US, UR, IS, and IR (four groups) represented the samples from uninfested rhizosphere soils, uninfected plant roots, infested rhizosphere soils, and infected plant roots, respectively. All these samples were immediately placed in sterile centrifuge tubes with sterile phosphate buffer saline (PBS) and divided into two parts for microbe analysis: one for culture-dependent method and another for culture-independent method.

*Meloidogyne incognita* was collected from the sponge gourd roots in Baiyun District, Guangzhou, China (113.27° N, 23.16° E) for the bioassay below and identified by SCAR marker (Song et al., 2019). The plant root was cut into 1–2 cm pieces, washed with NaClO (1%) for 3 min, then filtered by 100, 300, and 500 mesh screens, and the eggs collected from the 500 mesh were washed with sterile ultrapure water and incubated at 25°C for 5 days. The hatched pre-J2s were collected and washed three times with sterile ultrapure water, then sterilized with streptomycin sulfate solution (100 mg/mL) on a 100 rpm shaker at 25°C overnight. The pre-J2s were washed three times with sterile ultrapure water before use.

### 2.2. Culture-dependent microbial communities

Five grams of the rhizosphere soils from each sample were mixed and dissolved in 45 mL sterile PBS, and labeled as a start concentration (10^−1^ g/mL). Meanwhile, 5 g of the sterilized plant roots from each sample were ground with a sterile mortar, mixed with 45 mL sterile PBS, and also labeled as a start concentration (10^−1^ g/mL). The sample suspension was used to isolate bacteria and fungi on the plates of Trypticase soy agar (TSA; HKM, China), Potato dextrose agar (PDA), and Gause’s synthetic agar (GSA; HKM, China). The resulting plates were sealed with parafilm (BEMIS, United States) and cultured in the dark at 26°C. Nine plates were set up for each sample and each dilution level (root: 10^−1^, 10^−3^, 10^−5^, and 10^−7^ g/mL; soil: 10^−3^, 10^−5^, 10^−7^, and 10^−9^ g/mL). After 3 days, the bacterial and fungal colonies appeared on the plates were individually collected and inoculated into new plates. Microbe isolates were first grouped according to their colony and morphological characteristics. The resulting colonies were purified on the new plates and cultured in liquid LB (Lucia-Bertani; for bacteria) or PDA (for fungi) and kept at −80°C with 15% sterile glycerol. The DNA from each isolate was extracted using HiPure Bacterial DNA Kit and Fungal DNA Kit (Magen, China). The prokaryotic V4 region of bacterial 16S rRNA was amplified with primers 27F/1492R, and the ITS2 region of fungal ITS gene was amplified with ITS1F/ITS4R (Wu et al., 2020; Liu et al., 2021). The PCR products were extracted and then sequenced at the Beijing Genomics Institute (Guangzhou, China). The resulting sequences were compared with the data set in NCBI GenBank, and the representative isolates or clones for each bacterial or fungal species were submitted to GenBank (OP882106–OP882140; OP881962–OP881968).

### 2.3. Culture-independent microbial communities

The preparation of the samples was the same as that for the culture-dependent method. Total DNA from each sample was extracted and purified using the QIAamp DNA Stool Mini Kit (Qiagen, Hilden, Germany) according to the manufacturer’s instructions. After quantification by the NanoDrop ND-3300 spectrophotometer (NanoDrop Technologies, Thermo Scientific, Wilmington, DE, United States), PCR was carried out to generate amplicons in quintuplicate for rhizosphere soil samples and quadruplicate for plant root samples. The V5–V6 region of the bacterial 16S rRNA gene was amplified using primers 799F/1193R, and the ITS1 region of the fungal ITS gene was amplified using the primers BD-ITS1F/ITS2-2043R (Wu et al., 2020; Liu et al., 2021). All the primers contained a 12-bp barcode sequence at the 5′-end to distinguish the amplicons from different samples. The amplicons were equally combined to produce two separate PCR pools (bacterial and fungal amplicons separately), and then sequenced on an Illumina Nova 6000 platform (Guangdong Magigene Biotechnology, Guangzhou, China). The sequencing data were quality checked with Fastp (v. 0.14.1). Paired-end clean reads were merged using FLASH (v. 1.2.11) according to the relationship of the overlap between the paired-end reads (Magoè and Salzberg, 2011). Raw tags with at least 10 reads overlapping the opposite end of the same DNA fragment were merged (error ratio < 0.1). Sequences were assigned to each sample based on their unique barcodes. The barcodes and primers were removed before the clean tags were generated.

The OTUs were grouped with a threshold of 97% pairwise identity by Usearch software (v. 10.0.240). The most frequently occurring sequence was extracted as the representative sequence for each OTU and used for taxonomic annotation. The taxonomic classification was performed by SILVA (v. 119; Quast et al., 2013) and UNITE (v. 7.0; Kõljalg et al., 2013) for bacteria and fungi, respectively. The OTUs annotated as chloroplasts or mitochondria as well as not to the kingdom level, were removed. Based on the OTUs table, the unique, shared and core OTUs among four groups were illustrated by the UpSetR package using R software (Conway et al., 2017). The bacterial and fungal composition of the class, family, and genus levels for each group was calculated, and the histogram was drawn with the ggplot2 package using R software. The microbial alpha-diversity (Chao1 and Simpson index) analysis was performed by QIIME (v. 1.9.1; Caporaso et al., 2010), to measure the complexity of species diversity for a sample. Beta-diversity was used to evaluate the differences between samples in species complexity. Principal coordinate analysis (PCoA) based on euclidean distance matrixes was used for multivariate analysis of all samples, performed by the ggplot2 package of R software. Three non-parametric analyses (ANOSIM, analysis of similarity; Adonis, non-parametric multivariate analysis of variance; Amova, analysis of molecular variance) and one parametric analysis (Mrpp, multi-response permutation procedure) were performed by Vegan package of R software (v. 2.6–5, https://github.com/vegandevs/vegan/) and Mothur software (v. 1.35.1; Schloss et al., 2009), to test whether the differences among groups were significant (*p* < 0.05). A linear discriminant analysis (LDA) effect size (LEfSe) algorithm (Segata et al., 2011) was employed to identify the biomarkers for different groups, which was performed on the website.^1^ The value of LDA was used to estimate the effect size of each differentially abundant feature. Taxa with logarithmic LDA values over 4.00 were selected to perform histogram figures. The 16S rRNA and ITS amplicon data were deposited into the NCBI short-reads archive database under accession numbers PRJNA905571 and PRJNA905575, respectively.

### 2.4. Nematode dispersal assay

Thirty-three representative bacterial isolates from the culture-dependent communities were selected from the RKN-infested and uninfested groups for nematode dispersal assay (Supplementary Table 1). The bacteria were activated on LB plates, and the colonies were cultured in nutrient broth (agar; HKM, China) in a 120 rpm shaker at 25°C for 3 days. The dispersal of pre-J2s responding to the bacterial was tested according to the reported method (Wang et al., 2019). Briefly, the plates (9 cm diameter) were filled with 2% agarose and divided into three parts (Area A, Area B, and Area C, Supplementary Figure 2). For the J2 dispersal assay, 100 pre-J2s were placed in the middle of the plate (Area C), 50 μL of bacterial culture from different isolates according to the above-described culture method on Area A, and 50 μL of sterile nutrient broth or sterile extrapure water on Area B. After 4 h in the dark at 26°C, the nematodes in three areas were counted under a microscope, and the chemotactic index [CI = (the number of nematodes at Area A−the number of nematodes at Area C)/total number of nematodes] was calculated according to published methods (Wang et al., 2019). Each treatment was replicated four times. A CI ≥ 0.1 was considered attractive, a CI ≥ − 0.1 but <0.1 as a random response, and a CI < − 0.1 as repellent.

### 2.5. Nematicidal activity bioassay

The nematicidal activity of microbial isolates from culture-dependent microbial communities (Supplementary Table 12), was determined using the pre-J2s. The bacteria were activated on LB plates, and the colonies were cultured in nutrient broth in a 120 rpm shaker at 25°C for 3 days. The cultures were centrifuged at 12,000 rpm for 10 min at 4°C, and the resulting supernatants were filtered by 0.22 μm filters, and used to test for the bioassay of nematicidal activity. 100 pre-J2s in 80 μL sterile extrapure water and 20 μL streptomycin sulfate solution (100 mg/mL) were added to a well in the 48-well plate. The bacterial supernatants at concentrations of 100, 10, and 1%, which were prepared with sterile extrapure water, and were, respectively, introduced into the wells. Sterile nutrient broth or sterile extrapure water was used as the negative control. The plates were incubated at 25°C for 48 h, and the living and dead juveniles at 12, 36, and 48 h were counted under a microscope. Four replicates were established for each treatment. The experiment was repeated two times. Mortality was calculated according to the following formula: juvenile mortality = 100% × dead juveniles/total juveniles and the calibrated mortality rate = 100 × (mortality rate of strains−mortality rate of control)/(1−mortality rate of control) (Yin et al., 2021).

### 2.6. Statistical analysis

Statistical analysis was carried out by GraphPad Prism 5.0 software program (GraphPad Software, United States). Significant differences in nematicidal activity and nematode dispersal assays were determined by one-way ANOVA or Student’s *t*-test with a threshold of *p* < 0.05.

## 3. Results

### 3.1. Culture-dependent microbe communities

Overall, 32 bacterial species belonging to 23 genera from five classes, and eight fungal species belonging to five genera from five classes were identified from the soils and roots (Table 1). At the phylum level, 9, 3, 9, and 11 bacterial species from Actinobacteria, Bacteroidetes, Firmicutes, and Proteobacteria were identified, respectively. Seven and one fungal species were assigned to Ascomycota and Mucoromycota, respectively. Nine bacterial species (*Arthrobacter* sp., *Bacillus* sp., *B. ambifaria*, Enterobacteriaceae sp., *F*. *barbaricus*, *Microbacterium* sp., Micrococcaceae sp., Rhizobiaceae sp., and *Serratia* sp.) were shared, and *Arthrobacter* sp. and *Bacillus* sp. were dominant species, among all samples. On the contrary, no fungal species overlapped among the samples.

**Table 1:** Bacterial and fungal species detected by culture-dependent method.

Microbe species	Population sampled^*^			
	US	UR	IS	IR
**Bacteria**				
*Arthrobacter* sp.	++++	++	++++	++
*Bacillus* sp.	++++	+++	++++	+++
*Bacillus amyloliquefaciens*	++		++	
*Bacillus thuringiensis*	+		+	
*Burkholderia ambifaria*	+	+	+	+
*Chryseobacterium* sp.	+++	+	++++	
*Comamonas sediminis*	++	+	+	
*Cupriavidus metallidurans*		+		
Enterobacteriaceae sp.	++	+	+	+
*Ensifer adhaerens*	++		+++	
*Fictibacillus barbaricus*	++	+	+	+
*Flavobacterium* sp.	+		++	
*Gordonia* sp.		+		
*Leifsonia xyli*	+		+	
*Mammaliicoccus sciuri*	+			
*Massilia oculi*	+		+	
*Mesorhizobium* sp.	+++		++++	
*Microbacterium* sp.	++	+	++	+
*Microbacterium azadirachtae*	+			
Micrococcaceae sp.	+++	+	+++	++
*Paenibacillus* sp.		+		+
*Paenibacillus glycanilyticus*		++		++
*Priestia sp.*	+			
*Pseudomonas* sp.	+++		+++	
*Pseudomonas nitroreducens*			++	
Rhizobiaceae sp.	+	+	+	+
*Serratia* sp.	++	+	+	+
*Sphingobacterium puteale*	++		+++	
*Sporosarcina koreensis*		++		+
*Streptomyces* sp.	++			+
*Streptomyces viridobrunneus*		+		
*Terribacillus* sp.		+		
Total species	24	18	21	13
**Fungi**				
*Aspergillus* sp.				+
*Fusarium oxysporum*	++		+	
*Mucor irregularis*	+			
*Penicillium* sp.	++			
*Penicillium commune*	+			
*Penicillium desertorum*			+	
*Plectosphaerella cucumerina*			++	
*Sordariomycetes* sp.	+			
Total species	5	0	3	1

* +, ≤100 colonies detected in the plates; ++, > 100 ≤ 1,000 colonies detected in the plates; +++, > 1,000 ≤ 2,500 colonies detected in the plates; and ++++, >2,500 colonies detected in the plates.

Compared with the *M. incognita* infested samples, more bacterial colonies were detected from the uninfested samples, including root and soil samples (Table 1). Thirteen bacterial species (*B. amyloliquefaciens*, *B. thuringiensis*, *Ensifer adhaerens*, *Flavobacterium* sp., *Leifsonia xyli*, *Massilia oculi*, *M*. *sciuri*, *Mesorhizobium* sp., *M. azadirachtae*, *Priestia* sp., *Pseudomonas* sp., *P. nitroreducens*, and *Sphingobacterium puteale*) were detected only from soils (US and IS). In comparison, seven root-intrinsic (UR and IR) bacterial species were identified, including *Gordonia* sp., *C. metallidurans*, *Paenibacillus* sp., *P. glycanilyticus*, *Sporosarcina koreensis*, *S. viridobrunneus*, and *Terribacillus* sp. Compared with the uninfested soils, more colonies of *Chryseobacterium* sp., *E. adhaerens*, *Flavobacterium* sp., *Mesorhizobium* sp., and *S*. *puteale*, and litter colonies of *Comamonas sediminis*, *F*. *barbaricus*, and *Serratia* sp. were detected from the infested soils (Table 1). In addition, in the infested soils, *P. nitroreducens* was exclusively present, while *M*. *sciuri*, *M. azadirachtae*, *Priestia* sp., and *Streptomyces* sp. vanished after *M. incognita* infestation. In the root samples, little colonies of *S. koreensis* were observed after *M. incognita* infection, however, *Chryseobacterium* sp., *C*. *sediminis*, *Gordonia* sp., *C. metallidurans, S. viridobrunneus*, and *Terribacillus sp*. appeared only in the uninfected root samples while *Streptomyces* sp. only in the infected root samples.

For the fungi, *Penicillium desertorum* and *Plectosphaerella cucumerina* were exclusive in the infested rhizosphere soil, in contrast, *M. irregularis*, *Penicillium* sp. *Penicillium commune*, and *Sordariomycetes* sp. vanished in rhizosphere soils after *M. incognita* infestation. On the other hand, only one fungus, *Aspergillus* sp. was isolated from plant roots after the presence of *M. incognita*.

### 3.2. Microbial diversity of culture-independent communities

A total of 1,563,859 bacterial (16S rRNA) and 1,540,655 fungal (ITS) clean reads were obtained from 18 samples representing rhizosphere soils (US, IS) and plant roots (UR, IR). Each sample with over 96% of reads met the demand of Q30 (Supplementary Table 2). A total of 10,561 bacterial OTUs and 2,427 fungal OTUs were obtained (Supplementary Table 3). For bacteria, 555 genera belonging to 47 phyla, 105 classes, 204 orders, and 321 families were identified from 10,561 OTUs. For fungi, 192 genera belonging to 19 phyla, 39 classes, 84 orders, and 152 families were identified from 2,427 OTUs.

**Figure 1:**
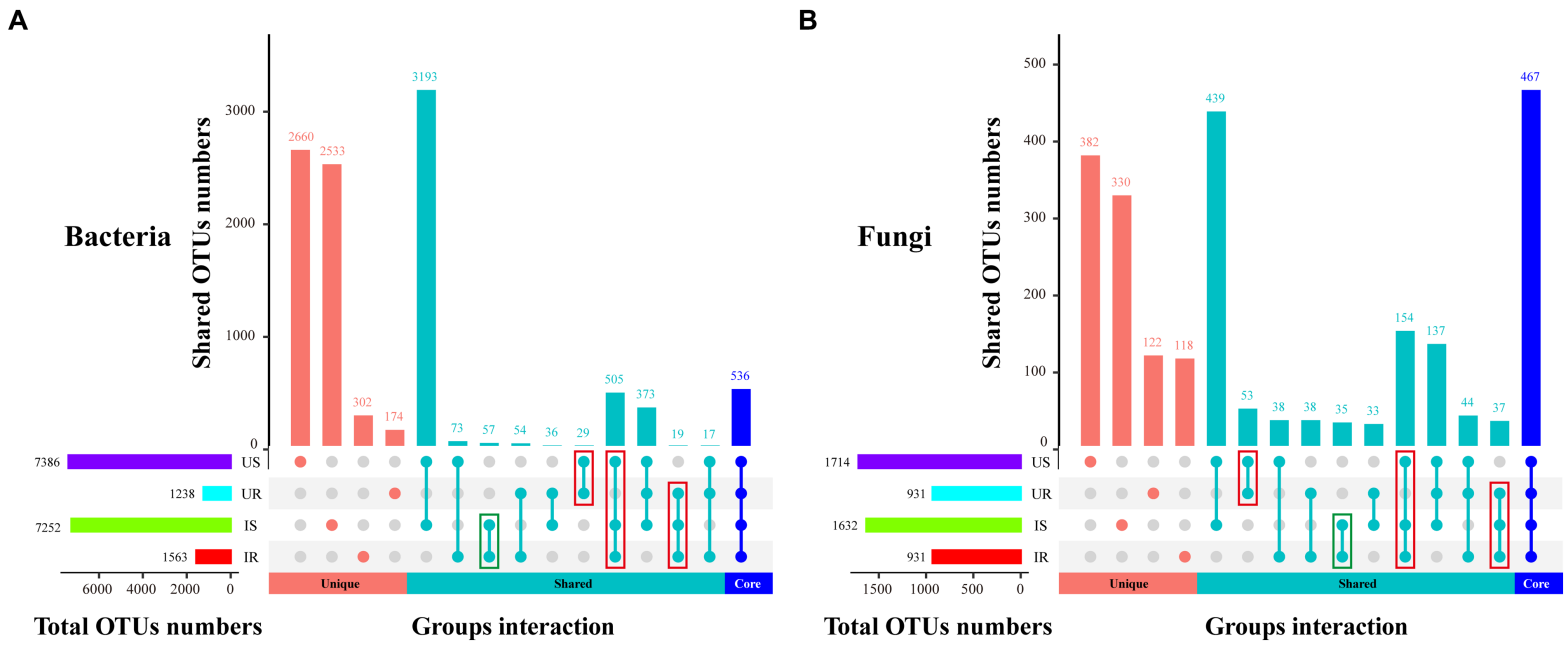
UpSetR plot depicts the unique, shared, core bacterial **(A)** and fungal **(B)** OTUs among four groups. The horizontal bar represents the total OTU numbers of each group, and the vertical bar represents the unique, shared, and core OTU numbers of different samples’ intersections. US, UR, IS, and IR represent the uninfested rhizosphere soil, uninfected plant root, infested rhizosphere soil, and infected plant root, respectively.

The number of bacterial OTUs in rhizosphere soils (US: 7386, IS:7252) was much more than in plant roots (UR: 1238, IR: 1563), and similar results were found in fungi (US:1714, IS: 1732, UR: 931, and IR: 931; Figure 1), indicating that the microbial species in rhizosphere soils were significantly higher than those in plant roots. A total of 536 bacterial and 467 fungal OTUs were shared in four groups (US, IS, UR, and IR), including 33 and 44 with more than 1% relative abundance in one or more groups such as *Ralstonia* sp., *Bacillus* sp., *Sphingomonas* sp. from bacteria, and *Thermomyces* sp., *Mortierella alpina*, *Penicillium simplicissimum*, and *Orbilia aurantiorubra* from fungi. The unique bacterial and fungal OTUs were detected in all groups but showed very low abundances. Five hundred and five bacterial and 154 fungal OTUs were shared between US, IS, and IR groups, suggesting these inherent soil OTUs may spread to the roots during *M. incognita* infestation. In contrast, the *M. incognita* infestation may also mediate the inherent root OTUs translocated to the soils, including 19 bacterial and 37 fungal OTUs shared in UR, IR, and IS groups. Furthermore, 57 bacterial and 35 fungal OTUs were specifically present in IS and IR. These results suggested that *M. incognita* may mediate the microbial exchange between rhizosphere soils and plant roots. On the other hand, 29 bacterial and 53 fungal OTUs vanished during the infestation, demonstrating the negative effect of *M. incognita* on microorganism communities (Figure 1; Supplementary Table 4).

The rarefaction curves for all samples showed that the sequence depths were reliable for both bacterial and fungal identification in each sample (Supplementary Figure 3). The apparent separation of rarefaction curves indicated the richness of bacteria and fungi in rhizosphere soils was significantly higher than that of the plant roots (Supplementary Figures 3A,C). The rank-abundant analysis showed that the bacterial evenness was similar in rhizosphere soil and plant root samples (Supplementary Figures 3B,D). However, the fungal evenness was lower in rhizosphere soils than in plant roots. In general, the diversity of bacteria and fungi was significantly higher in rhizosphere soils than that in plant roots.

Compared with UR and IR samples for the alpha-diversity analysis, significantly higher bacterial and fungal abundance were detected in US and IS, as indicated by the Chao1 index (Supplementary Figure 4; Supplementary Table 5). The Simpson index of bacteria in UR and IR were significantly higher than that in US (UR vs. US, *p* = 0.03) and IS (IR vs. IS, *p* = 0.035), while there was no significant difference in fungal infested groups (IR vs. IS, *p* = 0.123, Supplementary Table 6). Significantly higher diversity was present in bacterial (both US and IS) and fungal (US) rhizosphere soils, compared with the corresponding root groups (bacterial UR, IR, and fungal UR). On the other hand, the Chao1 (UR vs. US, *p* = 3.06 × 10^−6^) and Simpson (UR vs. US, *p* = 0.009) index of fungi in US were significantly higher than in UR, together with the rank-abundance results, showing more microbes species but less evenness in the uninfested soils than that in the uninfected roots. In addition, a similar Chao1 index (US vs. IS, *p* = 0.309) but a significantly higher Simpson index (US vs. IS, *p* = 0.033) in the fungal groups were detected in IS, compared with the US, suggesting that the infestation of *M. incognita* may decrease the fungal diversity in the soils.

**Figure 2:**
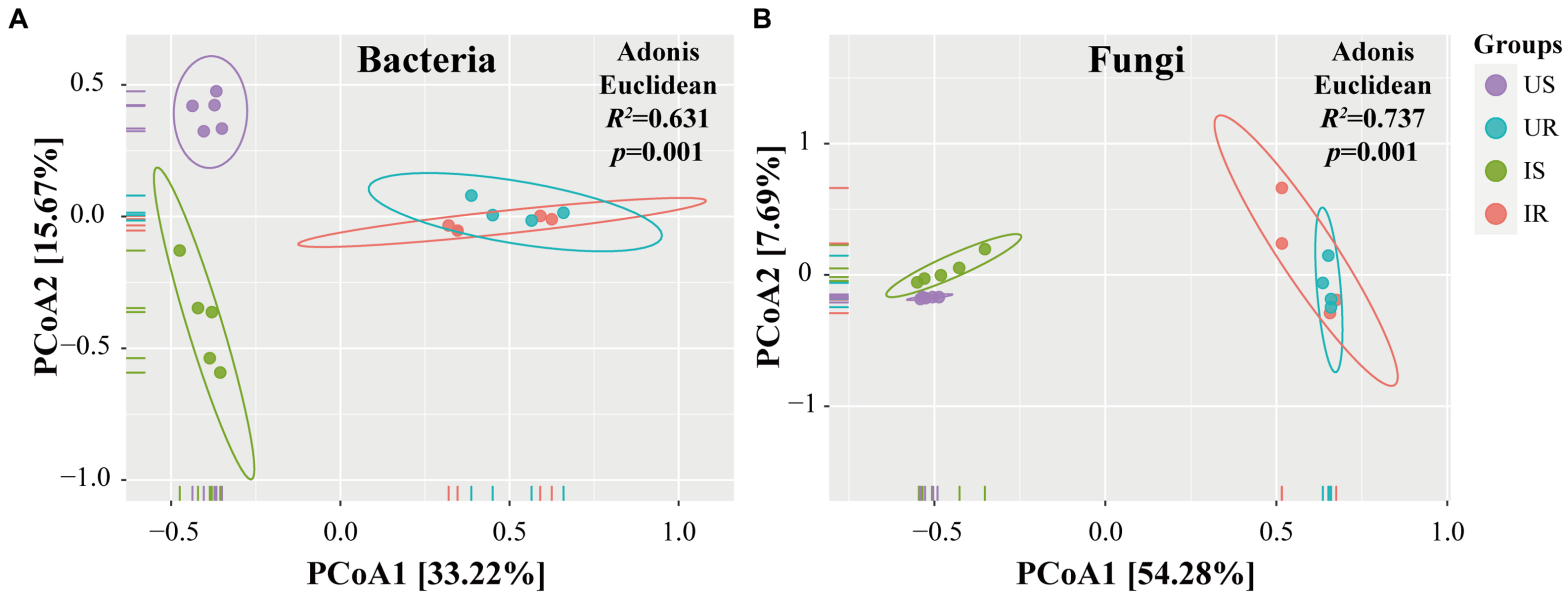
PCoA analysis of beta-diversity base on Euclidean distance matrix for bacteria **(A)** and fungi **(B)** communities of four groups. The Adonis was used to test the dissimilarity for bacteria (*R^2^* = 0.631, *p* = 0.001) and fungi (*R^2^* = 0.737, *p* = 0.001). US, UR, IS, and IR represent the uninfested rhizosphere soil, uninfected plant root, infested rhizosphere soil, and infected plant root, respectively.

To measure the extent of the similarity among microbial communities, we performed PCoA analysis on all rhizosphere soil and plant root samples, based on the Euclidean distance matrix (Figure 2). The bacterial communities among all samples could be divided into three groups (adonis, *R^2^* = 0.631, *p* = 0.001, Figure 2A). The microbial communities of the US samples were significantly different from the IS samples (*R^2^* = 0.361, *p* = 0.006) and two root samples (US vs. UR: *R^2^* = 0.674, *p* = 0.004; US vs. IR: *R^2^* = 0.558, *p* = 0.011), but no significant difference was observed between UR and IR samples (*R^2^* = 0.189, *p* = 0.098; Supplementary Table 7). Similar results were found in the fungal communities, and three groups were divided (*R^2^* = 0.737, *p* = 0.001, Figure 2B): US, IS, and root samples (UR and IR). Overall, microbial communities of the rhizosphere soil samples were significantly different from those of the plant root samples, and *M. incognita* infestation led to significant changes in the bacteria and fungi in rhizosphere soils.

**Figure 3:**
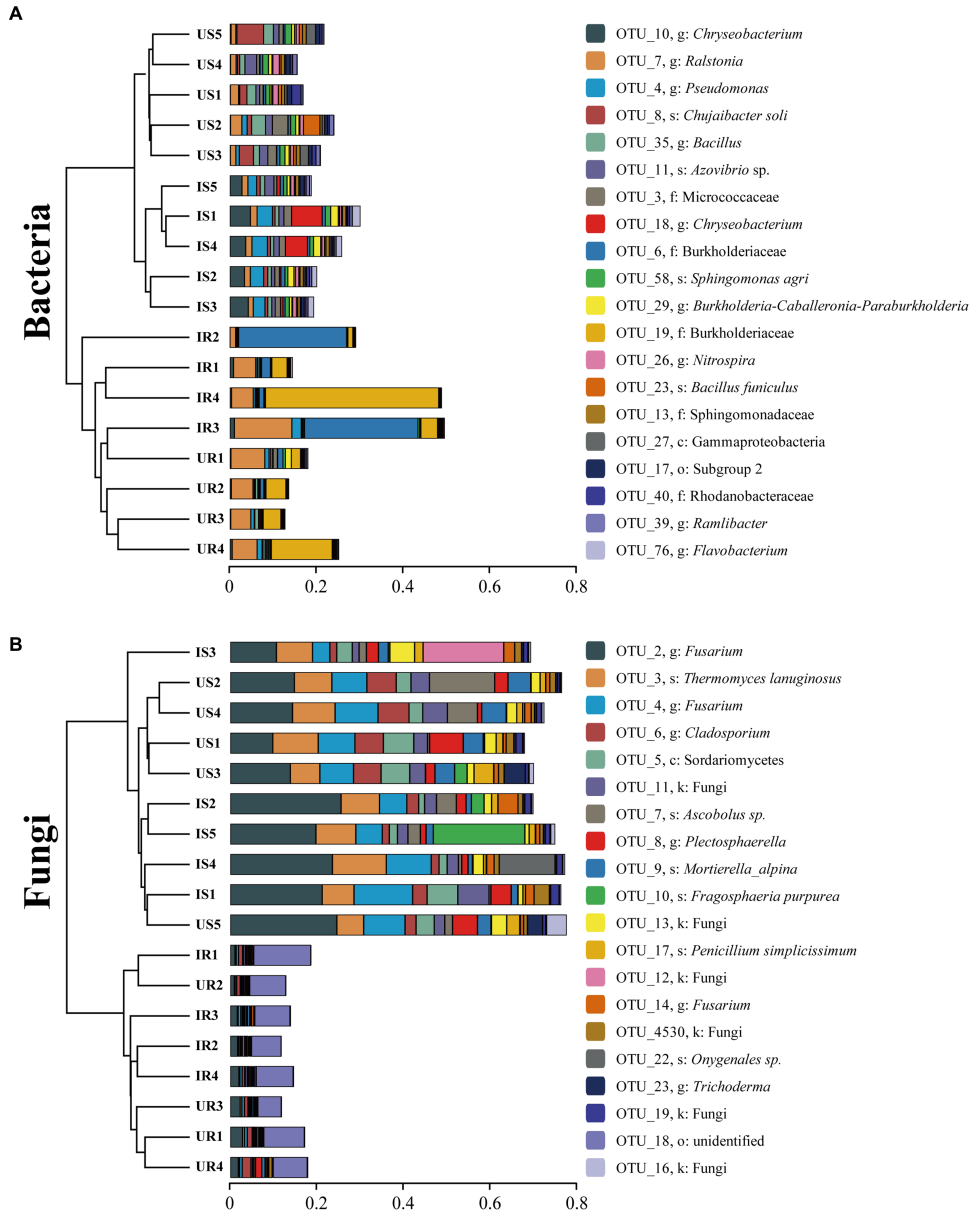
Clustering analysis of beta-diversity based on the Euclidean distance matrix for bacterial **(A)** and fungal **(B)** communities of all samples by UPGMA method. US, UR, IS, and IR represent the uninfested rhizosphere soil, uninfected plant root, infested rhizosphere soil, and infected plant root, respectively.

In addition, UPGMA clustering analysis was also done to evaluate the beta-diversity changes in four groups (Figure 3). The bacterial beta-diversities divided the samples into two parts: rhizosphere soil and plant root samples. When *M. incognita* nematodes were present in rhizosphere soil samples, the abundance of OTU_10 (*Chryseobacterium* sp.), OTU_76 (*Flavobacterium* sp.), and OTU_4 (*Pseudomonas* sp.) was significantly increased, while OTU_8 (*Chujaibacter soil*) and OTU_35 (*Bacillus* sp.) were decreased. In plant root samples, infestation with *M. incognita* significantly increased OTU_6 (Burkholderiaceae sp.). The fungal beta-diversities also clustered the samples into the same two parts as bacterial ones. The top 20 most abundant rhizosphere fungal OTUs accounted for more than 73% of total fungal abundance. However, the top 20 most abundant plant root endophytic fungal OTUs represented less than 15%, indicating the unevenness of the fungal communities in rhizosphere soils. Moreover, the infestation of *M. incognita* could significantly increase the abundance of OTU_2 (*Fusarium* sp.) and OTU_22 (*Onygenales* sp.) in the soil samples.

### 3.3. Bacterial and fungal population of culture-independent communities

The bacterial and fungal OTUs that increased or decreased after *M. incognita* invasion were listed in Supplementary Table 8. Compared with the increased bacterial OTUs, more decreased OTUs were identified (increase: 319; decrease: 646) in rhizosphere soils after *M. incognita* infestation. However, the decreased bacterial OTUs were only a quarter of the increased OTUs (181, 44) in plant root samples. On the other hand, the number of decreased fungal OTUs was higher than that of increased in rhizosphere soils (171, 278), while it was reversed in plant roots (166, 119). This implied that the endophytic microbes in plant roots were not adversely influenced by nematode invasion.

A total of 8, 15, 10, and 13 dominant bacterial OTUs (relative abundance higher than 1%) were identified from US, UR, IS, and IR groups, respectively (Supplementary Table 9). One bacterial species, OTU_7 (*Ralstonia* sp.), was the dominant OTU shared among four groups. Three dominant bacterial OTUs were shared by US and IS, while UR and IR shared eight dominant bacterial OTUs. Moreover, for the rhizosphere soil groups, the abundance of OTU_31 (*Myroides* sp.), OTU_1957 (*Flavobacterium* sp.), OTU_76 (*Flavobacterium* sp.), OTU_18 (*Chryseobacterium* sp.), and OTU_10 (*Chryseobacterium* sp.) in IS was at least 15 times higher than that in the US. On the contrary, OTU_8 (*Chujaibacter soli*), OTU_23 (*Bacillus* sp.), and OTU_27 (Gammaproteobacteria sp.) in the IS were suppressed to less than a quarter after *M. incognita* infestation (Supplementary Table 8). For plant root samples, the abundance of OTU_6 (Burkholderiaceae sp.), OTU_14 (*Pasteuria* sp.), and OTU_111 (uncultured bacterium) in IR significantly increased by at least 39 times compared with UR, while two Chitinophagaceae bacteria (OTU_150 and OTU_153) were dropped to less than a tenth. Specifically, the dominant OTU in UR, OTU_220 (*Xanthobacter flavus*), vanished after the *M. incognita* infestation (Supplementary Table 9).

A total of 14, 21, 19, and 18 dominant fungal OTUs were identified from US, UR, IS, and IR groups, respectively (Supplementary Table 9). OTU_2 (*Fusarium* sp.) was shared among four groups, especially with high relative abundance in soil samples (US: 15.62%, IS: 20.25%). Moreover, 10 dominant OTUs were shared in rhizosphere soils, and 13 dominant OTUs were shared in plant roots. For soil groups, seven OTUs significantly increased their abundance when *M. incognita* infestation. OTU_22 (*Onygenales* sp.) was 151 times more abundant in IS than in US groups. On the contrary, OTU_23 (Trichoderma sp.) decreased to a fifth in IS compared to US groups. For root groups, the abundance of OTU_50 (Ceratobasidiaceae sp.), OTU_281 (Fungi sp.), and OTU_321 (*O*. *aurantiorubra* sp.) was significantly increased after infestation, while OTU_49 (Rhizoctonia sp.), OTU_3063 (Rhizoctonia sp.) and OTU_126 (*Branch06*) was suppressed to less than one fifth (Supplementary Table 9).

**Figure 4:**
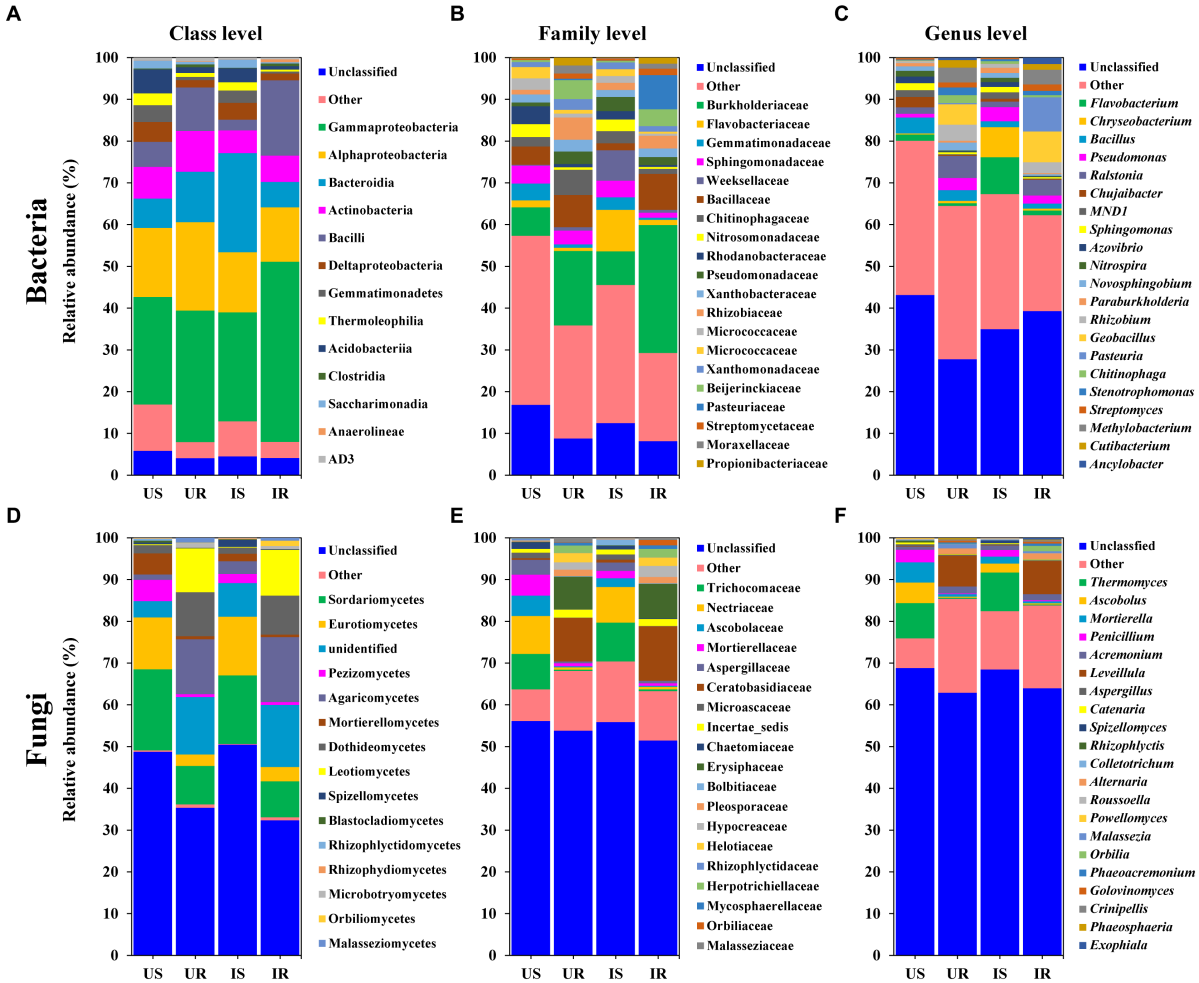
Relative abundance of the top 10 bacterial **(A–C)** and fungal **(D–F)** classes **(A,D)**, families **(B,E)**, and genera **(C,F)** in four groups. “Unclassified” represents the OTUs unassigned to the corresponding rank. “Others” includes classes, families, or genera beyond the top 10 OTUs. US, UR, IS, and IR represent the uninfested rhizosphere soil, uninfected plant root, infested rhizosphere soil, and infected plant root, respectively.

The top 10 bacterial taxa from different taxonomic levels of each group were defined as “major,” based on the relative abundance, including 13 at the class level, 20 at the family level, 21 at the genus level, and 28 at the species level (Figures 4A–C and Supplementary Table 10). For the class level, four bacterial classes shared in four groups were identified with more than 10% relative abundance in more than one group, including Gammaproteobacteria, Alphaproteobacteria, Actinobacteria, and Bacilli. At the family level, Burkholderiaceae was the major family with more than 6% relative abundance in all groups, and increased in plant roots after infestation (UR: 17.82%, IR: 30.65%). Another increased family in roots was Pasteuriaceae (0.33, 8.2%), 63 times in IR after infestation. Major bacterial genera were *Bacillus* and *Ralstonia*, with relative abundance >1% in each group. Most of the major bacterial species were uncultured, such as *Alpha proteobacterium*, *Rhizobiales* sp., and Xanthomonadaceae sp. (Supplementary Table 10).

The top 10 fungal taxa from each group included 15 from the class level, 19 from the family level, 21 from the genus level, and 23 from the species level (Figures 4D–F and Supplementary Table 10). The relative abundances of five major fungal classes were greater than 10% in one or more groups, including Sordariomycetes, Eurotiomycetes, Dothideomycetes, Agaricomycetes, and Leotiomycetes. At the family level, Nectriaceae and Trichocomaceae were the major fungal families in rhizosphere soils, and Ceratobasidiaceae and Erysiphaceae were major in plant roots. Major fungal genera included *Thermomyces* in rhizosphere soils and *Leveillula* in plant roots, with more than 7% relative abundance. The species of *M. alpina*, *Rozellomycota* sp., and Ceratobasidiaceae sp. shared among four groups, *Ascobolus* sp. (US: 4.88%, IS: 2.09%) and *P*. *simplicissimum* (2.38, 1.21%) shared between rhizosphere soil groups. *Onygenales* sp. (0.02, 2.58%) and *Spizellomyces* sp. (0.01, 0.68%) increased abundance in IS by more than 100 times compared with US, while *Agaricomycetes* sp. (0.24, 0.01%) was significantly suppressed to a twentieth in IS. On the other hand, *Helotiaceae* sp. (UR: 2.21%, IR: 1.97%), *Pleosporales* sp. (2.06, 0.99%), *Alternaria* sp. (1.36, 1.45%), and *Malassezia arunalokei* (0.81, 0.4%) existed in both root samples. After *M. incognita* infection, *Exophiala* sp. (0.003, 0.38%) increased 180 times higher in IR compared to UR, while *Branch06* sp. (1.43, 0.004%) was significantly decreased (Supplementary Table 10).

In addition to the major taxa above, the other taxa from different taxonomic levels that significantly varied after *M. incognita* invasion were also shown (Supplementary Table 11). For bacteria, 46 and 55 genera were increased dramatically in rhizosphere soils (such as *Elizabethkingia*, *Chryseobacterium*, *Myroides*, *Empedobacter*, and *Sphingobacterium*) and plant roots (such as *Pasteuria* and *Thiobacillus*), respectively. Meanwhile, 56 and 15 genera declined their abundance in soils (such as *Proteiniclasticum* and *Granulicella*) and roots (such as *Tistrella* and *Aquamicrobium*). Particularly, the abundance of four genera was altered differently in rhizosphere soils and plant roots. *Fluviicola* and *Peredibacter* increased their abundance in rhizosphere soils but decreased in plant roots, whereas *Deinococcus* and *Saccharopolyspora* were reversed. For fungi, 13 (37) and 21 (13) genera were significantly increasing (decreasing) their abundance in rhizosphere soils and plant roots, respectively. *Dactylella* was specifically present in the infested rhizosphere soils, while the entomopathogenic fungi *Beauveria bassiana* was significantly increased in IS. Remarkably, except for *Fusarium*, 36 out of 37 decreased genera in rhizospheric soils were not changed in roots, such as *Mortierella*, *Ascobolus*, and *Thielavia*. Detailed information on other taxonomic levels is shown in Supplementary Table 11.

### 3.4. Biomarkers for four groups

**Figure 5:**
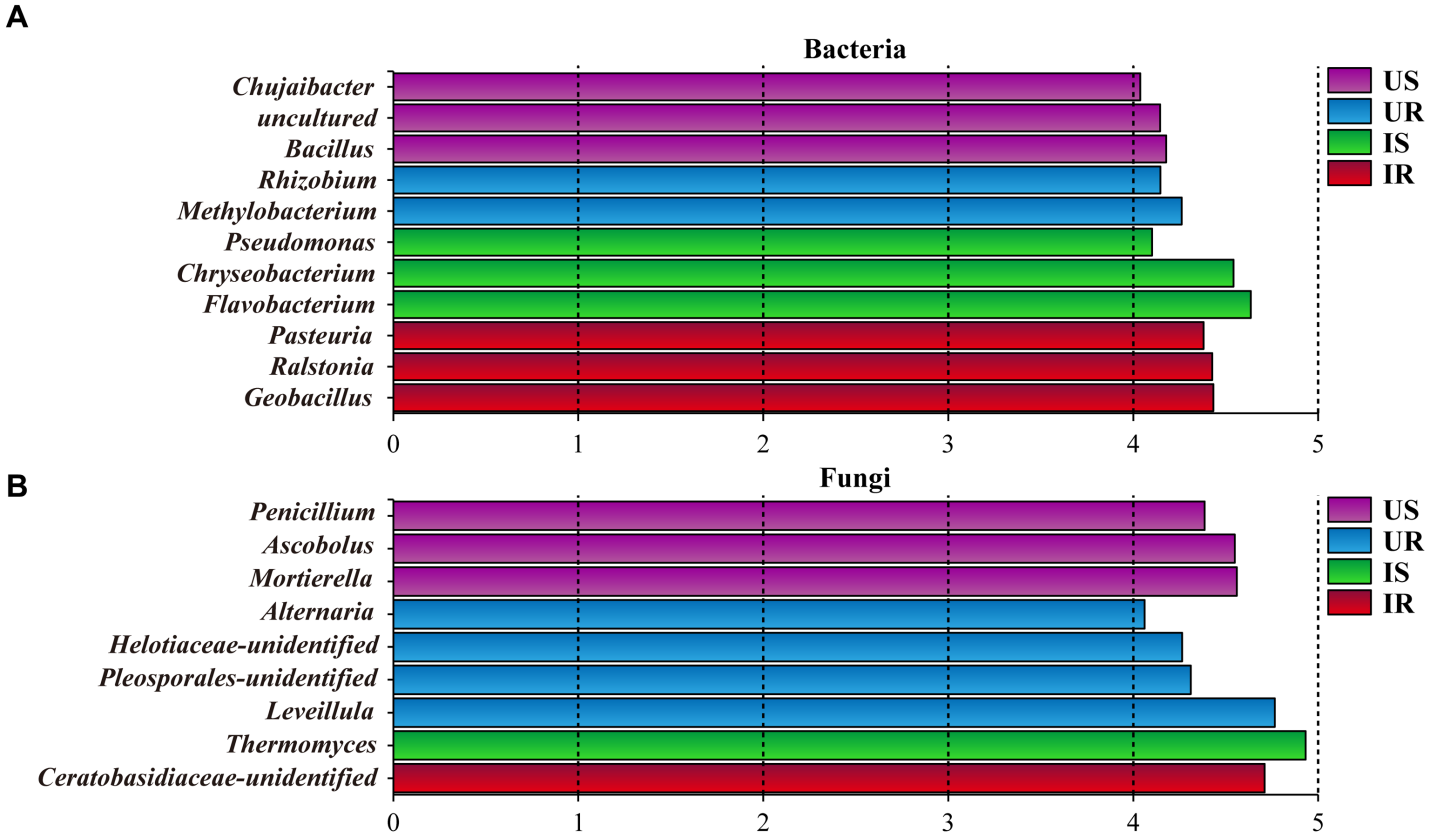
Histogram of discriminative bacterial **(A)** and fungal **(B)** genera among all samples. The genera with a logarithmic LDA value over 4 were selected. US, UR, IS, and IR represent the uninfested rhizosphere soil, uninfected plant root, infested rhizosphere soil, and infected plant root, respectively.

Rhizosphere soil and plant root samples were further compared with LefSe to determine the discriminative taxa affected by *M. incognita* infestation. A total of 52 bacterial taxa were identified as biomarkers (Logarithmic LDA > 4), including 22 from US, 10 from UR, 9 from IS, and 11 from IR. Exhibiting more discriminative taxa in uninfested rhizosphere soils than in infested soils. Genus *Bacillus* (Logarithmic LDA = 4.178, *p* = 0.021) was the most significant taxa in US, followed by *Chujaibacter* (LDA = 4.038, *p* = 0.004), while the genus *Flavobacterium* (LDA = 4.653, *p* = 0.002) had the largest effect sizes in IS. The most significant genus taxa in UR was *Methylobacterium* (LDA = 4.261, *p* = 0.004), and three genera with the largest effect sizes in IR were *Geobacillus* (LDA = 4.433, *p* = 0.005), *Ralstonia* (LDA = 4.426, *p* = 0.025), and *Pasteuria* (LDA = 4.380, *p* = 0.005; Figure 5A and Supplementary Table 12).

As to fungi, 53 taxa were selected as biomarkers, including 16, 16, 10, and 11 from US, UR, IS, and IR, respectively. Nine biomarkers were identified to be genus level, including *Penicillium* (LDA = 4.386, *p* = 0.004) and *Mortierella* (US, LDA = 4.559, *p* = 0.002) from US, which are species *P*. *simplicissimum* (LDA = 4.306, *p* = 0.003) and *M. alpina* (LDA = 4.549, *p* = 0.002) belonged; *Thermomyces* (LDA = 4.931, *p* = 0.004) from IS; *Leveillula* (LDA = 4.739, *p* = 0.005) and *Alternaria* (LDA = 4.033, *p* = 0.004) from UR (Figure 5B and Supplementary Table 12).

### 3.5. Nematode dispersal in responding to bacterial isolates

**Figure 6:**
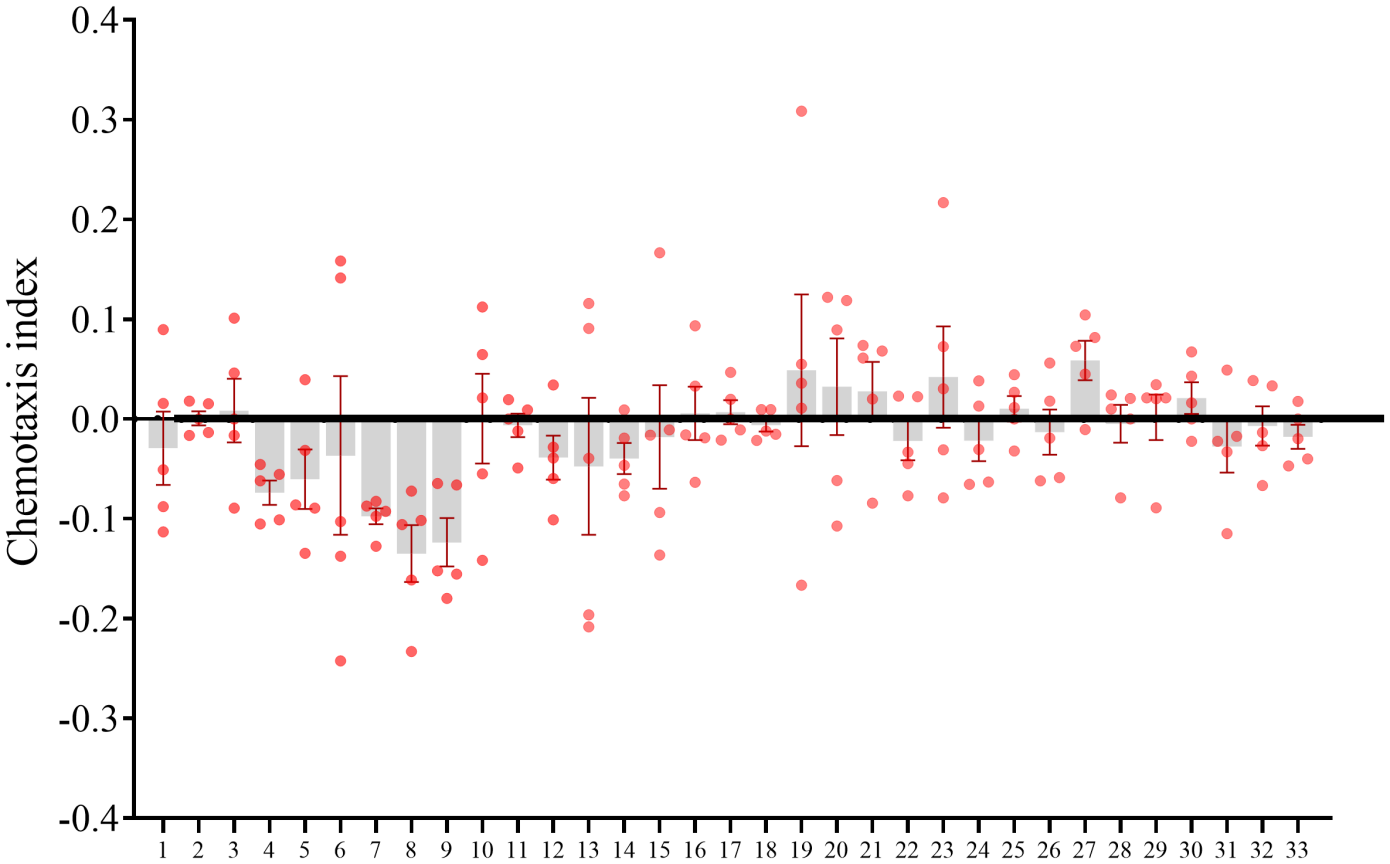
*Meloidogyne incognita* pre-parasitic second stage juveniles (pre-J2s) dispersal assay. The horizontal coordinates represent the numbers of culturable bacterial isolates.

The effects of bacterial isolates on *M. incognita* pre-J2s dispersal were tested. *B. amyloliquefaciens* from uninfested and infested rhizosphere soils, *Bacillus* sp. P35 from the uninfested rhizosphere soils, *M. azadirachtae* from the uninfested rhizosphere soils repelled nematodes (CI < −0.1), while no bacterial isolates attracted nematodes (CI > 0.1; Figure 6; Supplementary Table 13).

### 3.6. Nematocidal activity of bacterial isolates

**Figure 7:**
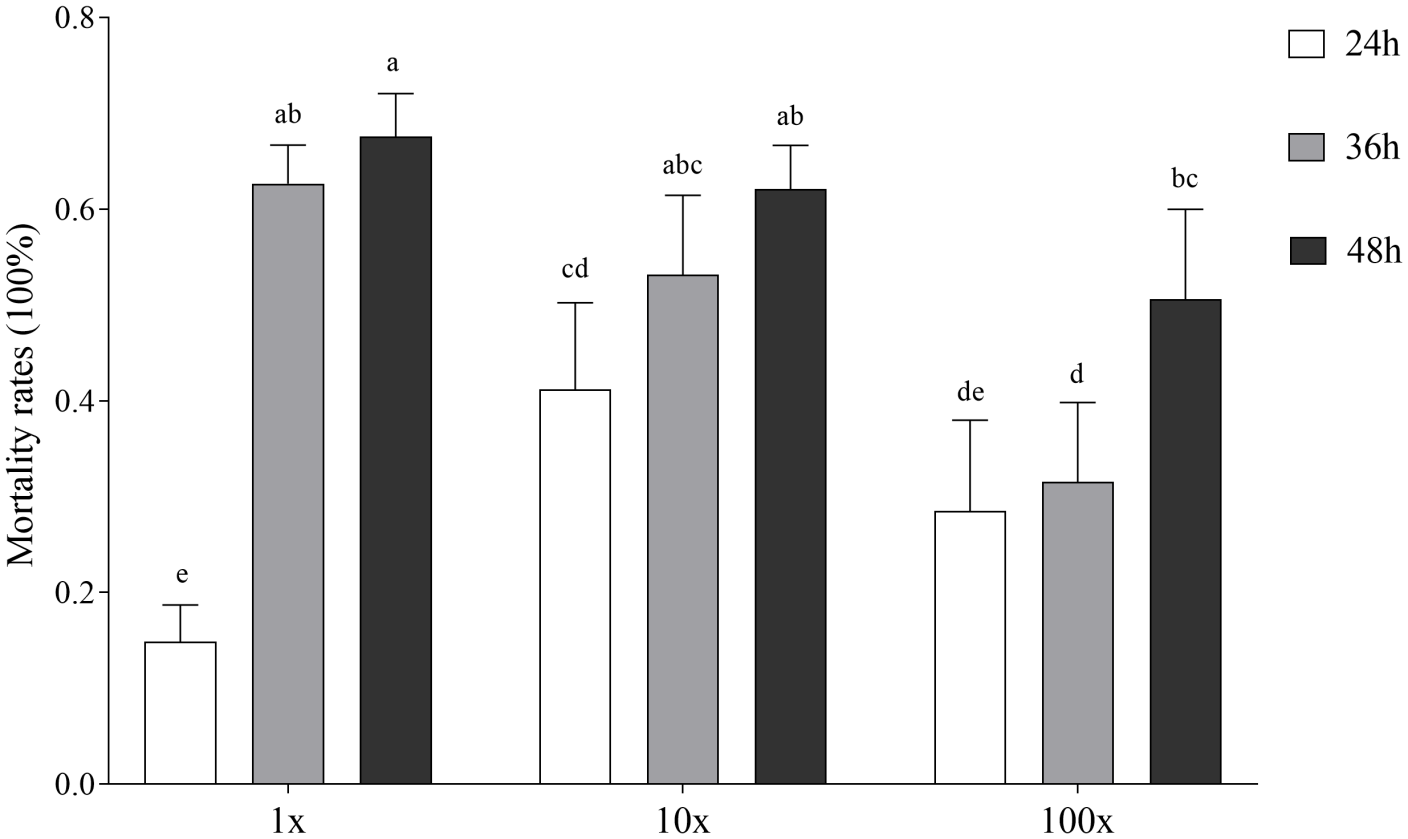
Effect of *Streptomyces* sp. TR27 culture supernatant on the calibrated mortality of *M. incognita* pre-parasitic second stage juveniles (pre-J2s)in different hours (24, 36, and 48 h). 1x, the supernatant of *Streptomyces* sp. TR27; 10x, the supernatant of *Streptomyces* sp. TR27 diluted 10 times; 100x, the supernatant of *Streptomyces* sp. TR27 diluted 100 times. Different letters indicated significant differences among different treatments (mean ± standard error, Tukey, *p* < 0.05).

*In vitro* bioassay results showed that the supernatant of *Streptomyces* sp. (isolate 27) from the infested roots (not present in the uninfected roots) showed the strongest toxicity to *M. incognita* pre-J2s at 48 h post incubation (Supplementary Table 14A). Compared with H_2_O and nutrient broth treatments, the supernatants of *Streptomyces* sp. TR27 significantly increased the mortality rates of pre-J2s at three concentrations (100, 10, and 1%) at 12, 36, and 48 h (Figure 7; Supplementary Tables 14B,C). It was also found that the nematocidal activity was correlated with the supernatant concentrations, with 67.59% calibrated mortality of pre-J2s in the original supernatant at 48 h.

## 4. Discussion

**Figure 8:**
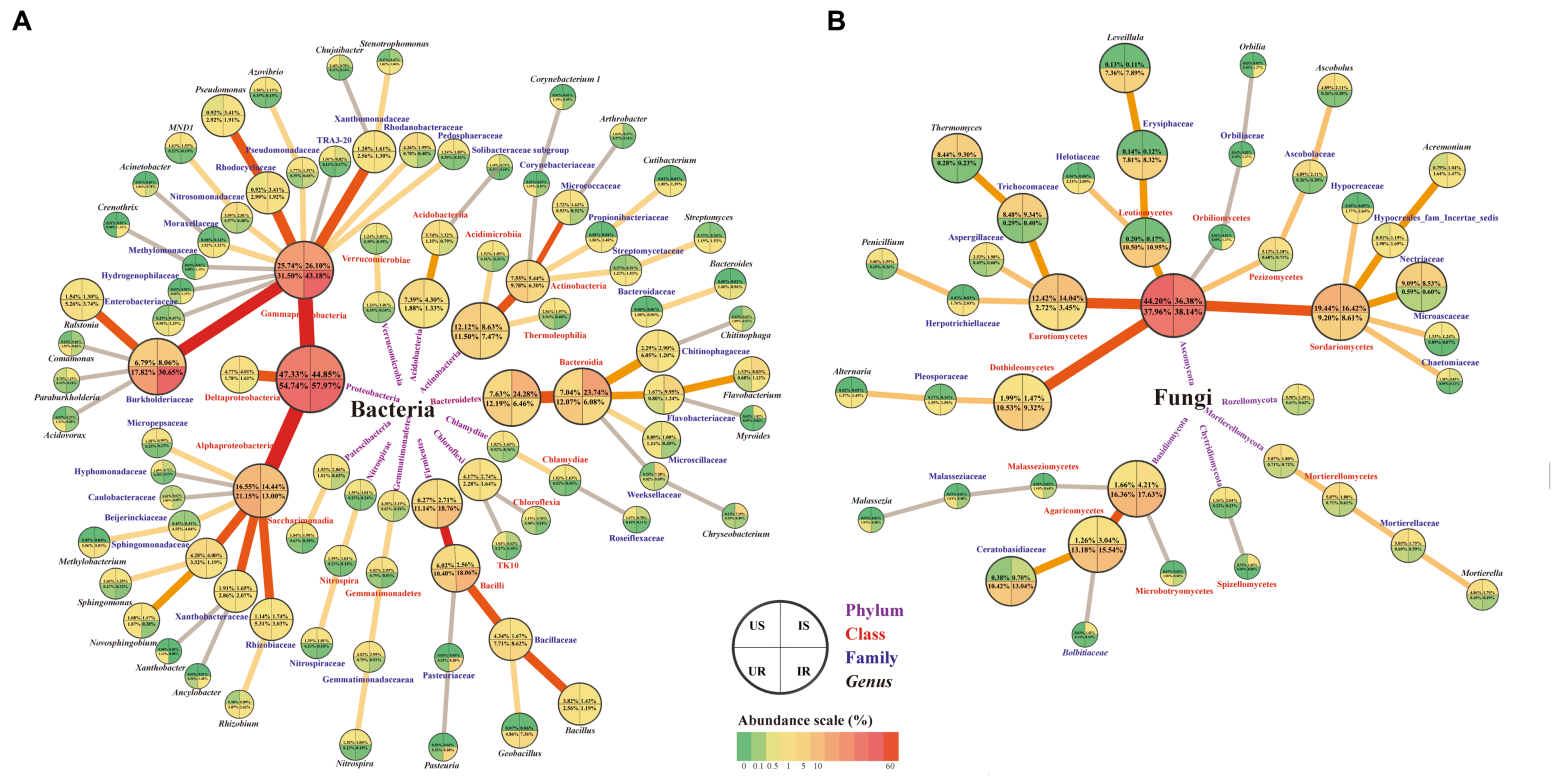
Summary of the OTU changes in bacterial **(A)** and fungal **(B)** community among four groups. Four taxonomic levels (phylum, class, family, and genus) were shown. The quarter circle represents corresponding samples according to the legend. Relative abundance is indicated by color gradients; the color from green through yellow to red represents the value from low to high. US, UR, IS, and IR represent the uninfested rhizosphere soil, uninfected plant root, infested rhizosphere soil, and infected plant root, respectively.

RKNs cause vital crop disease and huge loss of economic plants worldwide (Jones et al., 2013; Khan and Ahamad, 2020). Increasing evidences have demonstrated that the rhizosphere microbial community is indispensable in relieving nutrient stress and responding to pathogenic micro-invasion using root exudates from plant roots (Okubo et al., 2015). Specific resident rhizosphere soil and root-endophytic microbial communities that adapt to plants play essential roles in both optimizing growth and protecting against pathogen infection. The recruitment of beneficial microorganisms can also change the physiological function of plants to allow them to resist aerial pathogens (Kumar et al., 2012). In the present study, significant changes in the abundance of culturable and unculturable bacterial and fungal communities in rhizosphere soils and plant roots of sponge gourd were found to be associated with *M. incognita* invasion (Figure 8). Furthermore, among the detected bacterial isolates, *Streptomyces* sp. was discovered to exhibit nematocidal activity, and *B. amyloliquefaciens*, *Bacillus* sp. and *M. azadirachtae* to show repellent potentials for *M. incognita* pre-J2s, which can be used to develop RKN bio-control agents.

### 4.1. Effects of *Meloidogyne incognita* infestation on microorganisms in rhizosphere soils

RKNs significantly affect the rhizosphere soil and root-endophytic microbial communities of the host plant (Tian et al., 2015; Wolfgang et al., 2019; Zhou et al., 2019; Cao et al., 2022). Meanwhile, the microorganisms would produce secondary metabolites to affect the RKNs infection. In this study, 46 genera were significantly increased in rhizosphere soils after *M. incognita* infestation, including *Chryseobacterium*, *Elizabethkingia*, *Empedobacter*, and *Myroides*. Many of the species classified in these four genera are potential pathogens intrinsically resistant to a wide range of antibiotics. Most produce beta-lactamase and are often resistant to aminoglycosides, tetracyclines, chloramphenicol, erythromycin, clindamycin, and teicoplanin (Vandamme, 1994; Bellais et al., 2002; Vessillier et al., 2002; Kirby et al., 2004; Lin et al., 2019). *Sphingobacterium* was another genus increase in rhizosphere soils, including species *S*. *psychroaquaticum* (4.17 times) and *S. spiritivorum* (2.79 times). *Sphingobacterium psychroaquaticum* has the antagonistic ability on fungi pathogen, which could elicit the induced systemic resistance of the host plant (Xu et al., 2020). *Sphingobacterium spiritivorum* is sensitive to multiple antimicrobials and is considered resistant to many commonly administered antibiotics (Anthony and Verma, 2016; Gupta et al., 2016).

*Flavobacterium* spp. were essential in biological control by producing antibacterial effect factors and substances, extracellular macromolecular degrading enzymes, etc. Several root- and soil-derived *Flavobacterium* bacteria antagonize various phytopathogens and promote plant growth in different crops (Sang and Kim, 2012; Gunasinghe et al., 2015; Kwak et al., 2018; Carrión et al., 2019; Kraut-Cohen et al., 2021; Sun et al., 2021). Most of the *Pseudomonas* spp. show pathogens inhibitory activity attributing to iron sequestration *via* siderophore production, nutrient limitation or other unreported mechanisms, and some *Pseudomonas* species exhibit significant nematocidal activity and plant-growth promotion effects (Burlinson et al., 2013; Benner, 2014; Sun et al., 2021). In our study, the abundances of *Flavobacterium* (including *F*. *anatoliense*, *F. lindanitolerans*, *Flavobacterium* sp. GXW15-4, *F. ceti*, and *Flavobacterium* sp. MH51) and *Pseudomonas* bacteria in rhizosphere soils were 5.73 and 3.7 times higher after *M. incognita* infestation. It seemed that *Flavobacterium* and *Pseudomonas* bacteria in rhizosphere soils play special roles during *M. incognita* infestation, whether they have anti-nematode activity or plant growth ability need further research. On the contrary, genera *Arthrobacter*, *Bacillus*, and *Chujaibacter* decreased their abundance. *Bacillus* bacteria shows significant anti-nematode activity by producing plantazolicin (such as *B. amyloliquefaciens* FZB42) or inducing systemic resistance in plants (such as *B. atrophaeus* GBSC56) (Liu et al., 2013; Xiong et al., 2015; Cheng et al., 2020; Ayaz et al., 2021; Migunova et al., 2021; Yin et al., 2021). Moreover, *Bacillus* colonization was negatively correlated with pathogen abundance and disease incidence (Xue et al., 2015). Consistently, in the present results, the abundance of *Bacillus* bacteria (US vs. IS: 3.82% vs. 1.43%) in rhizosphere soils significantly decreased after *M. incognita* infestation. Interestingly, two *Bacillus* species (*B. amyloliquefaciens* and *Bacillus* sp.) isolated by culture-dependent method repelled *M. incognita* pre-J2s. Why *M. incognita* presence decreased the abundance of *Bacillus* species which can repel the nematodes needs further investigation. In addition, *M. azadirachtae,* a plant growth-promoting actinobacterium (Madhaiyan et al., 2010), which repelled nematodes from the uninfested rhizosphere soils. Therefore, it may be a potential root-knot nematode control agent, which is worthy of further development.

RKN infection of plants leads to physiological changes in the host and affects the composition of root exudates (Tian et al., 2015). Some of these root exudates can directly antagonize disease and insect pests, while others can collect and select microbial flora conducive to plant growth under the action of microbial chemotaxis (Bücking et al., 2008; Cao et al., 2022). It is suggested that the different regulations of the abundance of *Bacillus*, *Flavobacterium*, and *Pseudomonas* resulted from the interaction among nematodes, fungi, and plants in rhizosphere soils. However, all of these bacterial genera were potential nematicides. *Chujaibacter soli* is essential in polyfluoroalkyl substances and lindane degradation, while *Arthrobacter* sp. can degrade unusual and polymeric compounds (Senevirathna et al., 2022; Wu et al., 2022). Both bacteria play a crucial role in biodegrading agrochemicals and pollutants but were suppressed by *M. incognita* in rhizosphere soils. Indicating *M. incognita* not only hindered plant growth but also damaged the recovery ability of soil from pollution.

*Dactylella* fungi comprise 72 species, all of which are considered to trap and consume nematodes (Ahrén et al., 1998). *Dactylella* sp. was specifically present in rhizosphere soils after *M. incognita* infestation, and it appeared this fungus might come along with the nematode. In addition, the abundance of genus *Beauveria*, especially *B*. *bassiana*, was increased in rhizosphere soils after *M. incognita* infestation. *Beauveria bassiana* (Bb) is a filamentous fungus that can secrete a variety of extracellular enzymes and it is a well-known biological insecticide, including nematicidal activity (Kepenekci et al., 2017; Amobonye et al., 2020). Three major fungal genera including *Catenaria*, *Mortierella*, and *Penicillium* (mainly *P*. *simplicissimum*) were suppressed in rhizosphere soils after *M. incognita* infestation. Species of *Catenaria* are known primarily as pathogens of nematodes (Platzer and Platzer, 1999; Castillo and Lawrence, 2011). Some species from *Mortierella* and *Penicillium* can produce antibiotic and has potential antagonist activity against various plant pathogens (Platzer and Platzer, 1999; Tagawa et al., 2010). *P. simplicissimum* can release nematicidal alkaloid such as peniprequinolone A and penigequinolone B (Kusano et al., 2000). They were suppressed to less than a half in the rhizosphere soils after *M. incognita* infestation, which was confirmed by the culture-dependent method. Notably, *Pisolithus* vanished in infested rhizosphere soils, known for producing antibiotics such as pisolithins A and B (Kope et al., 1991). In the infested rhizosphere soils, the composition and abundance of these anti-pathogenic fungi changed oppositely, which may result from the competitive effects of nematodes toxin and plant root exudates.

### 4.2. Effects of *Meloidogyne incognita* infestation on plant root-endophytic microorganisms

Six root-endophytic bacterial genera significantly increased their abundance after *M. incognita* infestation, but no bacterial genera significantly decreased in this process. The bacterial genus or species decreased in infested rhizosphere soil, such as *Bacillus*, *C. soli*, and *Arthrobacter* were not changed in the infected plant root, indicating the plant root could protect the root-endophytes. Genus *Flavobacterium* increased 4.2 times in the infected roots. It appears to be a widespread bacterium (present at rhizosphere soils and plant roots) that plays a protective role from plant pathogenic infection. Some species of genera *Geobacillus* and *Pasteuria* in rhizosphere soils or plant roots show insecticidal or nematicidal activity, such as *G. thermoglucosidasius* and *P. penetrans* (Liu et al., 2019; Siddharthan et al., 2022). Both genera were not changed at rhizosphere soils but significantly increased in the infected roots, especially genus *Pasteuria*. Similarly, *Ancylobacter*, *Methylobacterium*, and *Streptomyces* increased in infected plant roots. All these three genera play essential roles in plant growth promotion (Fu et al., 2021; Worsley et al., 2021; Nie et al., 2022). Moreover*, Streptomyces* display enhanced antagonistic activities and suppression of the root rot and wilt diseases in pulses (Manikandan et al., 2022). It is speculated that the internal root environment specifically increased the population of beneficial bacteria.

On the other hand, we found three major root-endophytic fungal genera increased abundance after infection, including *Exophiala*, *Orbilia*, and *Phaeoacremonium*. Many species in the genus *Orbilia* have been confirmed to be knob-forming nematophagous hyphomycetes, which could capture the nematodes utilizing adhesive stalked knob (Liu et al., 2005). This indicated that a high abundance of *O*. *aurantiorubra* might be essential in protecting the *L. cylindrica* from *M. incognita* infection. Moreover, *Fusarium mangiferae* and *Pestalotiopsis* spp. disappeared in the infected roots, both of which can secrete cytotoxins (Hamzah et al., 2018; Zhao et al., 2021). All these bacterial and fungal beneficial plant growth promoters or antagonists with diverse anti-nematode activity in roots became dominant after *M. incognita* infection, demonstrating the protective effect of the plant on these genera.

In summary, in the case of *M. incognita* invasion, microbial communities in rhizosphere soils were mainly affected by two factors, the secondary metabolites secreted by *M. incognita* and the exudates secreted by plant root. The secreta from *M. incognita* may increase the abundance of bacteria with antibiotic-resistant activity (such as *Chryseobacterium*, *Elizabethkingia*, *Empedobacter*, *Myroides*, and *Sphingobacterium*), but suppress the fungi with antibiotic activity (*Ascobolus, M. alpina*, and *P. simplicissimum*), as well as increase the bacteria associate with reducing soils’ ability to recover from pollutions (such as *Chujaibacter* and *Arthrobacter*). Meanwhile, in response to the *M. incognita* invasion, the root exudates may provide a better environment to promote the growth of nematode eaters or plant growth promotors (such as *Flavobacterium*, and *Pseudomonas* from bacteria, *Dactylella* and *Beauveria* from fungi). On the other hand, in the infected plant roots, the host plant trended to protect the beneficial bacteria that were suppressed in the infested soil (such as *Bacillus*, *Chujaibacter*, and *Arthrobacter*), and increased the abundance of insecticidal or nematicidal (*F. lindanitolerans*, *Flavobacterium.* sp. GXW15-4, *F. ceti*, *Flavobacterium*. sp. MH51, and *F. anatoliense*, *Geobacillus*, *Pasteuria*, and *Streptomyces* from bacteria, *Orbilia* from fungi) and plant growth-promoting (*Streptomyces* and *Methylobacterium*) bacteria. And may be due to the enhancement of the immune system, toxin-secreting fungi (*F. mangiferae* and *Pestalotiopsis*) were eliminated in the infected plant roots. More candidate microorganisms are listed in Supplementary Table 10, which is worthy further study.

## 5. Conclusion

In this study, from the surrounding soils and roots of sponge gourd with and without *M. incognita*, 32 bacterial and 8 fungal isolates were detected by culturable method. Ten thousand five hundred and sixty-one unculturable bacterial and 2,427 fungal OTUs were also obtained. Supernatant of *Streptomyces* sp. from the uninfected soils exhibited approximately 67.59% nematocidal activity against *M. incognita* pre-J2s. *B. amyloliquefaciens*, *Bacillus* sp., and *M. azadirachtae* repelled the nematodes. *M. incognita* invasion significantly influenced the microbiota composition in rhizosphere soils and plant roots. These results provided insights into exploring novel microbiological agents for safe control of these harmful plant nematodes.

## Data availability statement

The datasets presented in this study can be found in online repositories. The names of the repository/repositories and accession number(s) can be found in the article/Supplementary material.

## Author contributions

RH designed and coordinated the research. LQ, LC, and JW collected the soil and root samples. LQ and KD collected the eggs and pre-J2s of *Meloidogyne incognita*. LQ conducted the research and wrote the manuscript. LQ and ZR analyzed the data. RH and ZR revised the manuscript. All authors contributed to the article and approved the submitted version.

## Funding

The work was supported by the GDAS Special Project of Science and Technology Development (2022GDASZH-2022010101) and the Guangzhou Science and Technology Project (202206010120).

## Conflict of interest

The authors declare that the research was conducted in the absence of any commercial or financial relationships that could be construed as a potential conflict of interest.

## Publisher’s note

All claims expressed in this article are solely those of the authors and do not necessarily represent those of their affiliated organizations, or those of the publisher, the editors and the reviewers. Any product that may be evaluated in this article, or claim that may be made by its manufacturer, is not guaranteed or endorsed by the publisher.

## Footnotes

^1^ http://cloud.magigene.com/
