# Supplementary material for: Microbial landscapes of the rhizosphere soils and roots of Luffa cylindrica plant associated with Meloidogyne incognita
Source: Front Microbiol. 2023 May 25;14:1168179. doi: 10.3389/fmicb.2023.1168179 (PMC10247985; doi:10.3389/fmicb.2023.1168179)
Supplement: Supplementary file 1 [file Table_1.DOCX]

Supplementary Table 1. List of 33 culturable bacterial isolates used for nematocidal and dispersal bioassay.

| Number | Isolate | Name | **IS** | **IR** | **UR** | **US** |
| --- | --- | --- | --- | --- | --- | --- |
| 1 | P8 | Micrococcaceae sp. | √ |  |  | √ |
| 2 | G19 | *Microbacterium* sp. | √ | √ | √ | √ |
| 3 | G4 | *Ensifer adhaerens* | √ |  |  | √ |
| 4 | P11 | *Chryseobacterium sp.* | √ |  |  | √ |
| 5 | P14 | *Bacillus* sp. | √ |  |  | √ |
| 6 | P27 | *Priestia* sp. |  |  |  | √ |
| 7 | P33 | *Bacillus amyloliquefaciens* | √ |  |  | √ |
| 8 | P35 | *Bacillus* sp. |  |  |  | √ |
| 9 | PR6 | *Microbacterium azadirachtae* |  |  |  | √ |
| 10 | T1 | *Mesorhizobium* sp. | √ |  |  | √ |
| 11 | T10 | *Pseudomonas nitroreducens* | √ |  |  |  |
| 12 | T17 | *Comamonas sediminis* | √ |  | √ | √ |
| 13 | T24 | *Massilia oculi* | √ |  |  | √ |
| 14 | T28 | Enterobacteriaceae sp. | √ | √ | √ | √ |
| 15 | T3 | *Sphingobacterium puteale* | √ |  |  | √ |
| 16 | T36 | *Mammaliicoccus sciuri* |  |  |  | √ |
| 17 | T7 | *Pseudomonas* sp. | √ |  |  | √ |
| 18 | TR10 | *Cupriavidus metallidurans* |  |  | √ |  |
| 19 | TR12 | *Streptomyces viridobrunneus* |  | √ | √ | √ |
| 20 | TR15 | *Paenibacillus glycanilyticus* |  | √ | √ |  |
| 21 | T2 | *Metabacillus indicus* |  | √ | √ | √ |
| 22 | TR20 | Rhizobiaceae sp. | √ | √ | √ | √ |
| 23 | TR21 | *Paenibacillus* sp. |  | √ | √ |  |
| 24 | TR22 | *Terribacillus* sp. |  |  | √ |  |
| 25 | TR23 | *Bacillus* sp. |  | √ | √ | √ |
| 26 | TR24 | *Streptomyces* sp. |  |  |  | √ |
| 27 | TR27 | *Streptomyces* sp. |  | √ |  |  |
| 28 | P10 | *Arthrobacter* sp. | √ | √ | √ | √ |
| 29 | TR7 | *Gordonia* sp. |  |  | √ |  |
| 30 | TR11 | *Sporosarcina koreensis* |  | √ | √ |  |
| 31 | T20 | *Fictibacillus barbaricus* | √ | √ | √ | √ |
| 32 | PR17 | *Bacillus altitudinis* | √ | √ | √ | √ |
| 33 | T33 | *Serratia* sp. | √ |  |  | √ |
