# Supplementary material for: Microbial landscapes of the rhizosphere soils and roots of Luffa cylindrica plant associated with Meloidogyne incognita
Source: Front Microbiol. 2023 May 25;14:1168179. doi: 10.3389/fmicb.2023.1168179 (PMC10247985; doi:10.3389/fmicb.2023.1168179)
Supplement: Supplementary file 13 [file Table_13.DOCX]

Supplementary Table 13. Effects of bacterial isolates on *M*. *incognita dispersal*.

| Strain | Name | CI1 | CI2 | CI3 | CI4 | CI5 | Average |
| --- | --- | --- | --- | --- | --- | --- | --- |
| 1 | Micrococcaceae sp. | 0.09 | -0.09 | 0.02 | -0.11 | -0.05 | -0.03 |
| 2 | *Microbacterium* sp. | -0.02 | -0.01 | 0.02 | 0.00 | 0.02 | 0.00 |
| 3 | *Ensifer adhaerens* | 0.10 | -0.02 | 0.00 | 0.05 | -0.09 | 0.01 |
| 4 | *Chryseobacterium sp.* | 0.08 | 0.11 | 0.06 | 0.16 | -0.12 | 0.06 |
| 5 | *Bacillus sp.* | -0.09 | -0.13 | -0.09 | -0.03 | 0.04 | -0.06 |
| 6 | *Priestia sp.* | 0.16 | 0.14 | -0.14 | -0.24 | -0.10 | -0.04 |
| 7 | *Bacillus amyloliquefaciens* | -0.12 | -0.16 | -0.06 | -0.14 | -0.25 | -0.14 |
| 8 | *Bacillus* sp. P35 | -0.16 | -0.17 | -0.22 | -0.17 | -0.16 | -0.17 |
| 9 | *Microbacterium azadirachtae* | -0.32 | -0.28 | -0.34 | -0.34 | -0.31 | -0.32 |
| 10 | *Mesorhizobium* sp. | -0.05 | -0.14 | 0.06 | 0.02 | 0.11 | 0.00 |
| 11 | *Pseudomonas nitroreducens* | 0.00 | 0.01 | -0.05 | -0.01 | 0.02 | -0.01 |
| 12 | *Comamonas sediminis* | -0.06 | -0.21 | -0.02 | -0.04 | 0.08 | -0.05 |
| 13 | *Massilia oculi* | -0.04 | -0.21 | 0.12 | -0.20 | 0.09 | -0.05 |
| 14 | Enterobacteriaceae sp. | 0.07 | 0.33 | -0.21 | 0.00 | 0.06 | 0.05 |
| 15 | *Sphingobacterium puteale* | 0.17 | -0.09 | -0.01 | -0.02 | -0.14 | -0.02 |
| 16 | *Mammaliicoccus sciuri* | 0.09 | -0.06 | 0.03 | -0.02 | -0.02 | 0.01 |
| 17 | *Pseudomonas* sp. | -0.11 | -0.06 | -0.04 | -0.09 | 0.03 | -0.06 |
| 18 | *Cupriavidus metallidurans* | -0.10 | 0.11 | -0.09 | -0.17 | -0.13 | -0.08 |
| 19 | Streptomyces viridobrunneus | 0.01 | 0.04 | 0.31 | 0.06 | -0.17 | 0.05 |
| 20 | *Paenibacillus glycanilyticus* | -0.06 | -0.11 | 0.09 | 0.12 | 0.12 | 0.03 |
| 21 | *Metabacillus indicus* | 0.06 | 0.02 | 0.07 | 0.07 | -0.08 | 0.03 |
| 22 | Rhizobiaceae sp. | 0.12 | 0.11 | 0.02 | -0.05 | 0.07 | 0.05 |
| 23 | *Paenibacillus* sp. | 0.07 | -0.08 | 0.03 | 0.22 | -0.03 | 0.04 |
| 24 | *Terribacillus* sp. | -0.07 | -0.03 | -0.06 | 0.04 | 0.01 | -0.02 |
| 25 | *Bacillus* sp. | 0.04 | -0.03 | 0.01 | 0.00 | 0.03 | 0.01 |
| 26 | *Streptomyces* sp. | -0.06 | 0.06 | 0.02 | -0.06 | -0.02 | -0.01 |
| 27 | *Streptomyces* sp. | -0.14 | 0.01 | -0.14 | -0.15 | -0.04 | -0.09 |
| 28 | *Arthrobacter* sp. | 0.00 | 0.02 | 0.01 | -0.08 | 0.02 | 0.00 |
| 29 | *Gordonia* sp. | -0.09 | 0.02 | 0.03 | 0.02 | 0.02 | 0.00 |
| 30 | *Sporosarcina koreensis* | -0.01 | -0.12 | -0.02 | -0.04 | -0.09 | -0.06 |
| 31 | *Fictibacillus barbaricus* | 0.05 | -0.02 | -0.11 | -0.03 | -0.02 | -0.03 |
| 32 | *Bacillus altitudinis* | 0.04 | -0.07 | -0.03 | 0.03 | -0.01 | -0.01 |
| 33 | *Serratia* sp. | -0.04 | -0.05 | 0.00 | -0.02 | 0.02 | -0.02 |

1. Dispersal of *M. incognita* second-stage juvenile.
2. Strains with repeated CI ≥ 0.05 and CI < −0.05 (A).

| Strain | Name | CI1 | CI2 | CI3 | CI4 | CI5 | Strain |
| --- | --- | --- | --- | --- | --- | --- | --- |
| 5 | *Bacillus* sp. | -0.06 | -0.06 | -0.10 | -0.11 | -0.05 | -0.07 |
| 7 | *Bacillus amyloliquefaciens* | -0.09 | -0.09 | -0.08 | -0.10 | -0.13 | -0.10 |
| 8 | *Bacillus* sp. P35 | -0.07 | -0.16 | -0.11 | -0.23 | -0.10 | -0.13 |
| 9 | *Microbacterium azadirachtae* | -0.18 | -0.15 | -0.16 | -0.06 | -0.07 | -0.12 |
| 10 | *Mesorhizobium* sp. | -0.05 | -0.14 | 0.06 | 0.02 | 0.11 | 0.00 |
| 12 | *Comamonas sediminis* | -0.03 | -0.04 | 0.03 | -0.10 | -0.06 | -0.04 |
| 14 | Enterobacteriaceae sp. | -0.08 | -0.07 | -0.02 | 0.01 | -0.05 | -0.04 |
| 17 | *Pseudomonas* sp. | 0.00 | 0.05 | 0.02 | -0.02 | -0.01 | 0.01 |
| 18 | *Cupriavidus metallidurans* | 0.01 | 0.01 | -0.02 | -0.01 | -0.02 | -0.01 |
| 22 | Rhizobiaceae sp. | 0.02 | 0.02 | -0.08 | -0.03 | -0.04 | -0.02 |
| 27 | *Streptomyces* sp. | 0.07 | 0.04 | -0.01 | 0.08 | 0.10 | 0.06 |
| 30 | *Sporosarcina koreensis* | -0.02 | 0.00 | 0.07 | 0.04 | 0.02 | 0.02 |
