# Supplementary material for: Microbial landscapes of the rhizosphere soils and roots of Luffa cylindrica plant associated with Meloidogyne incognita
Source: Front Microbiol. 2023 May 25;14:1168179. doi: 10.3389/fmicb.2023.1168179 (PMC10247985; doi:10.3389/fmicb.2023.1168179)
Supplement: Supplementary file 14 [file Table_14.DOCX]

**Supplementary Table 14. Effect of *Streptomyces* sp. TR27 supernatants on the mortalities of *M. incognita*.**

1. Mortality rates of *M. incognita* pre-parasitic second stage juveniles (pre-J2s) by *Streptomyces* sp. TR27 supernatants at 48 h.

| Strain | Isolates | Dilutions | Mortality rates | | | | Average |
| --- | --- | --- | --- | --- | --- | --- | --- |
| H2O | H2O | ×1 | 1.41% | 5.19% | 3.45% |  | 3.35%±0.02 |
| NB | Nutrient Broth | ×1 | 27.16% | 35.96% | 25.96% | 23.96% | 28.26%±0.05 |
| 27 | *Streptomyces* sp. TR27 | ×1 | 83.13% | 80.00% | 71.13% | 80.95% | 78.80%±0.05 |
|  |  | ×10 | 78.72% | 68.42% | 72.53% | 64.20% | 70.97%±0.06 |
|  |  | ×100 | 70.45% | 69.81% | 61.90% | 62.96% | 66.28%±0.04 |

1. Mortality rates of *M. incognita* pre-parasitic second stage juveniles (pre-J2s) by *Streptomyces* sp. TR27 supernatants.

| Strain | Dilution times | Hours | Mortality rates | | | | Average |
| --- | --- | --- | --- | --- | --- | --- | --- |
| *Streptomyces* sp. | ×100 | 24h | 38% | 41% | 25% | 24% | 31.91%±0.05 |
| TR27 |  | 36h | 52% | 49% | 48% | 37% | 46.48%±0.03 |
|  |  | 48h | 62% | 59% | 63% | 74% | 64.49%±0.03 |
|  | ×10 | 24h | 51% | 52% | 40% | 34% | 43.98%±0.04 |
|  |  | 36h | 73% | 58% | 60% | 63% | 63.37%±0.03 |
|  |  | 48h | 75% | 71% | 76% | 69% | 72.73%±0.02 |
|  | ×1 | 24h | 23% | 21% | 15% | 16% | 18.90%±0.02 |
|  |  | 36h | 66% | 73% | 73% | 71% | 70.80%±0.02 |
|  |  | 48h | 80% | 76% | 73% | 78% | 76.69%±0.02 |
| NB | ×1 | 24h | 5% | 5% | 4% | 5% | 4.73%±0.00 |
|  |  | 36h | 19% | 22% | 25% | 21% | 21.80%±0.01 |
|  |  | 48h | 26% | 27% | 33% | 27% | 28.07%±0.02 |
| H_2_0 | ×1 | 24h | 2% | 3% | 5% | 3% | 3.15%±0.01 |
|  |  | 36h | 9% | 5% | 5% | 8% | 6.53%±0.01 |
|  |  | 48h | 16% | 9% | 8% | 13% | 11.52%±0.02 |

C.The calibrated mortality rate of *M. incognita* pre-J2 by *Streptomyces* sp. TR27 supernatants.

| Dilution times | Hours | Calibrated mortality rate |
| --- | --- | --- |
| ×100 | 24h | 28.53%±0.05 de |
|  | 36h | 31.56%±0.04 d |
|  | 48h | 50.64%±0.05bc |
| ×10 | 24h | 41.20%±0.05cd |
|  | 36h | 53.16%±0.04abc |
|  | 48h | 62.09%±0.02ab |
| ×1 | 24h | 14.88%±0.02e |
|  | 36h | 62.66%±0.02ab |
|  | 48h | 67.59%±0.02a |
