# Supplementary material for: Microbial landscapes of the rhizosphere soils and roots of Luffa cylindrica plant associated with Meloidogyne incognita
Source: Front Microbiol. 2023 May 25;14:1168179. doi: 10.3389/fmicb.2023.1168179 (PMC10247985; doi:10.3389/fmicb.2023.1168179)
Supplement: Supplementary file 15 [file Data_Sheet_1.docx]

Supplementary Material

# Supplementary Figures and Tables

## Supplementary Figures


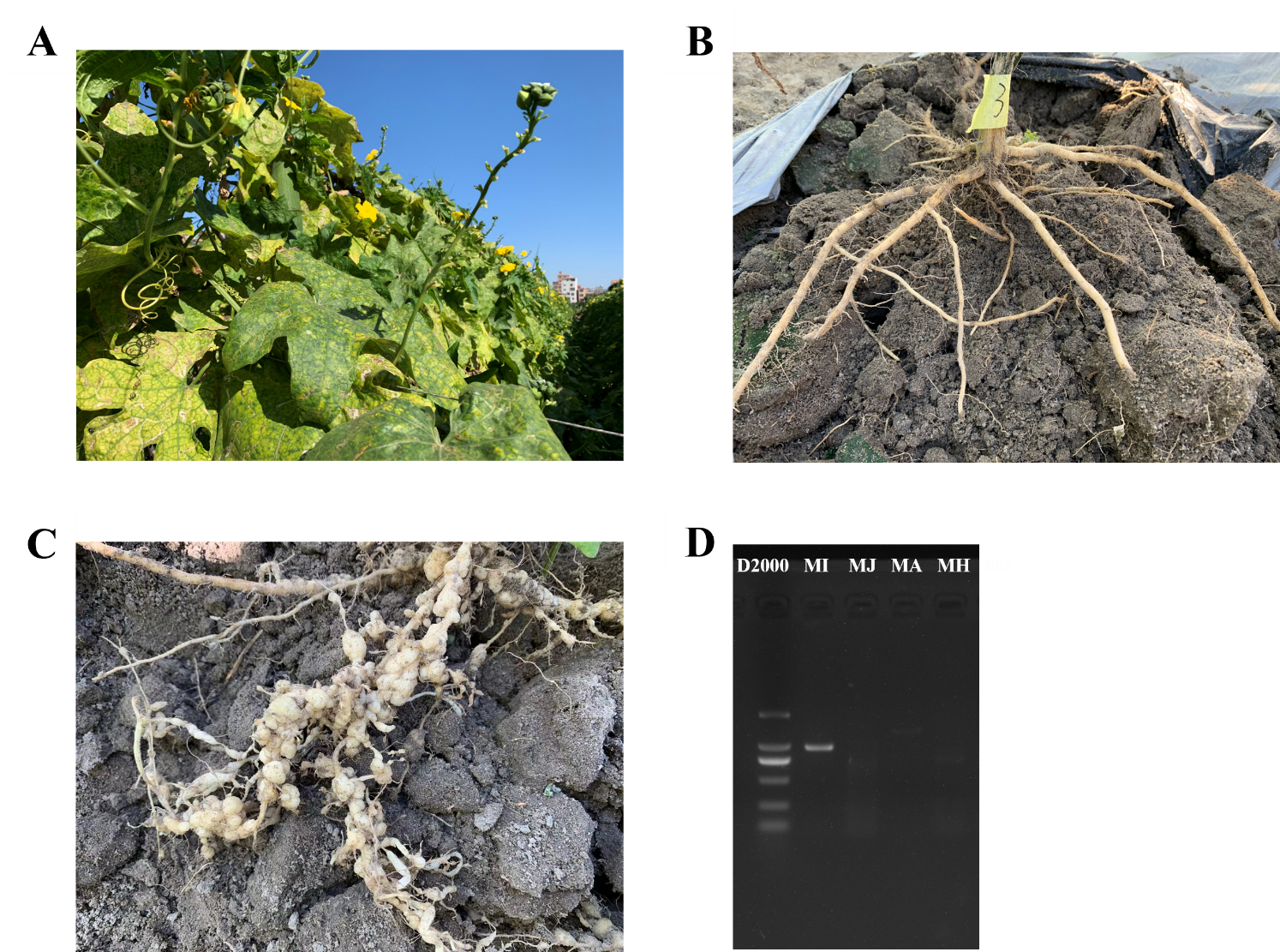


**Supplementary Figure 1**. the root of the sponge gourd and molecular identification of nematodes. A. sponge gourd (*Luffa cylindrica*); B. the healthy root of sponge gourd; C. the infected root of sponge gourd; D. molecular identification of nematodes. MI, *M. incognita*; MJ, *M. javanica*; MA, *M. arenaria*; MH, *M. hapla*.


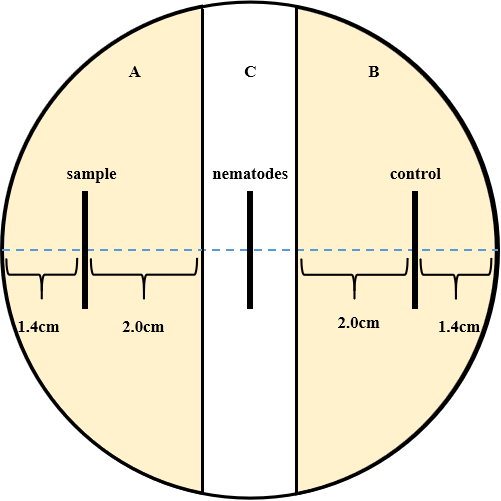


**Supplementary Figure 2.** Methods for nematode dispersal index in a 9 cm Petri dish with agarose (2%). A, the area for bacterial supernatant; B, the area for sterile nutrient broth or sterile extrapure water; and C, the area for nematodes.


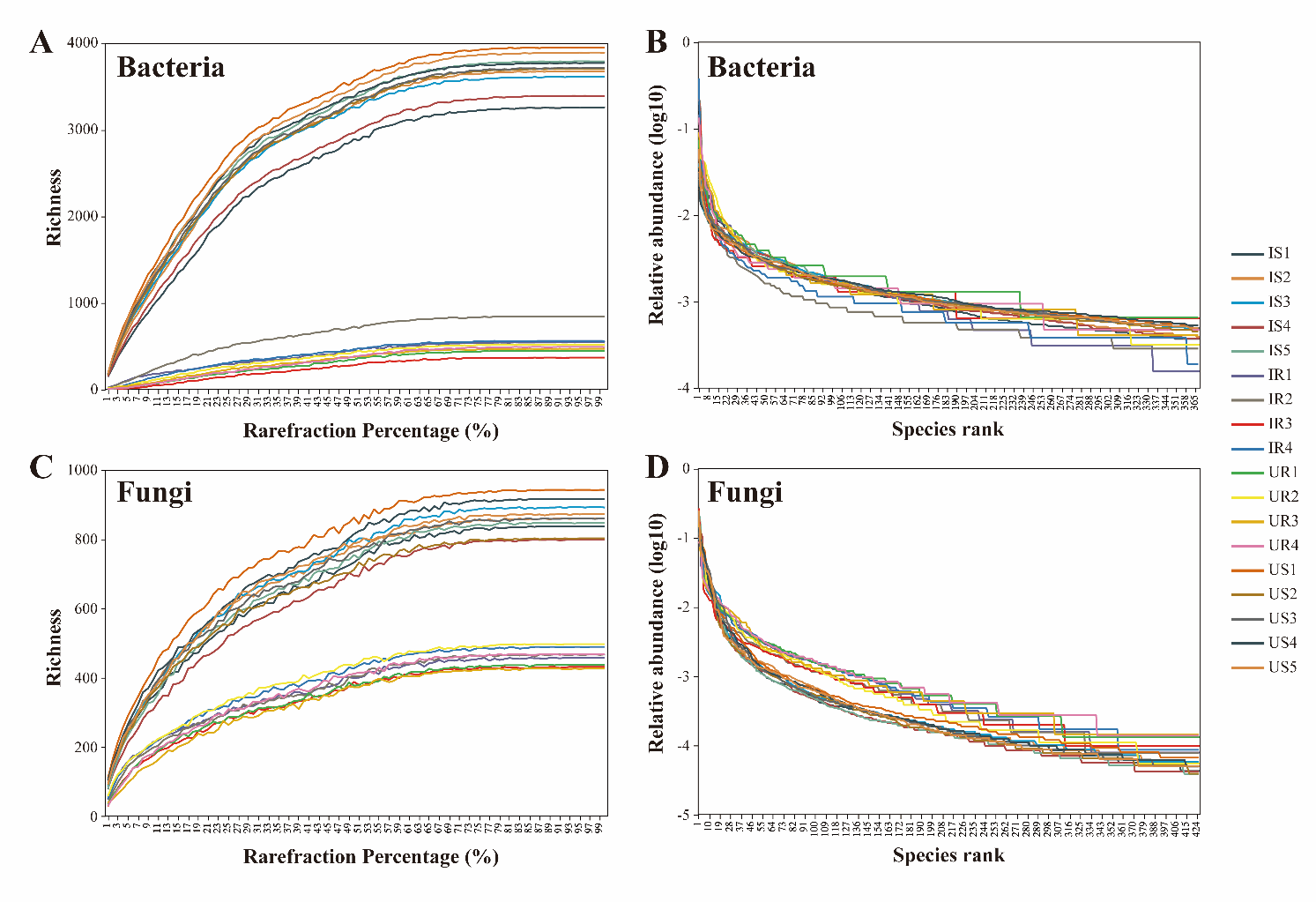


**Supplementary Figure 3.** Rank-abundance and rarefaction curves for all samples. Rarefaction curves for bacteria (A) and fungi (C); Rank-abundance curves for bacteria (B) and fungi (D).


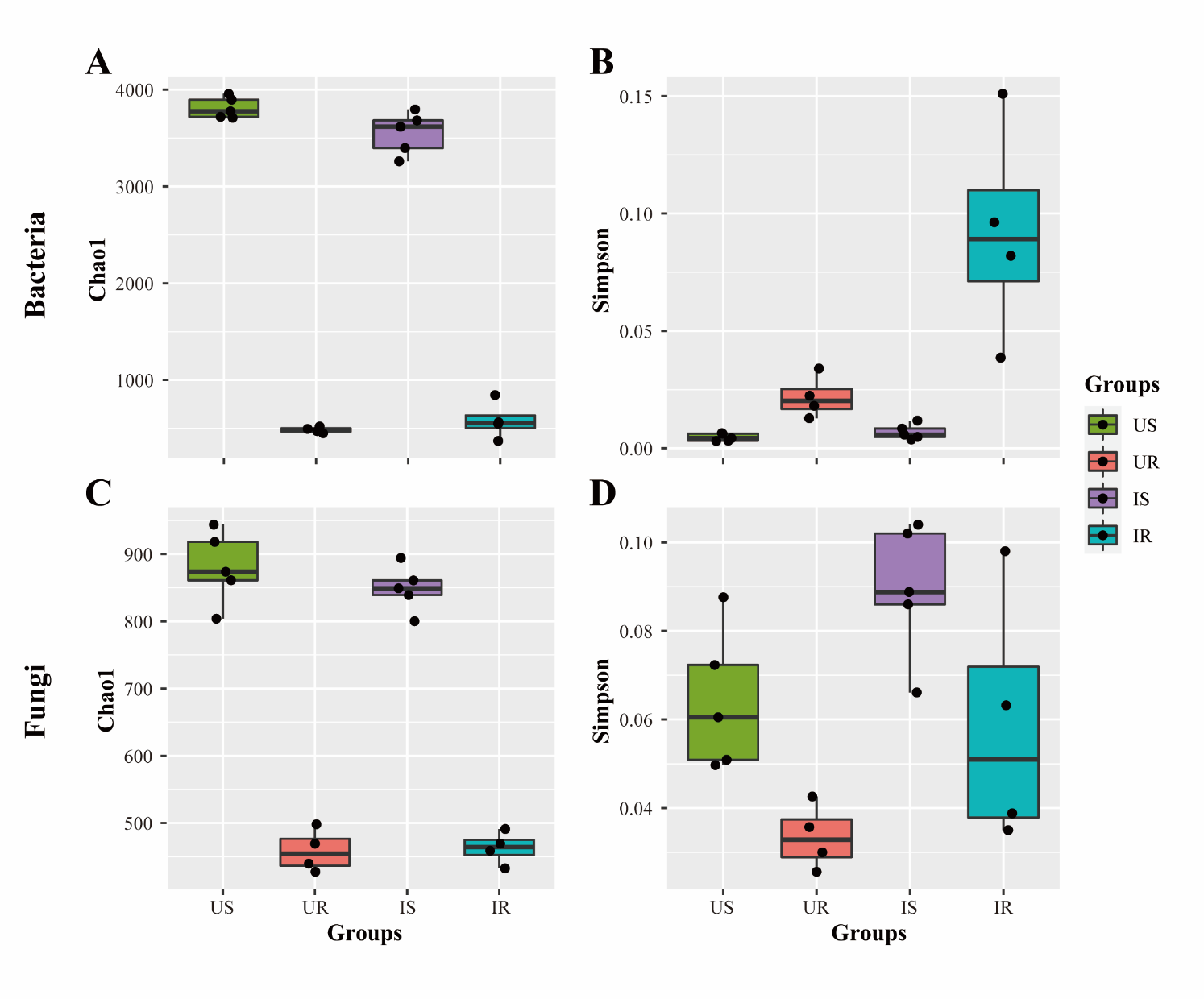


**Supplementary Figure 4.** Boxplot of Chao1 and Simpson indices of bacterial (A, B) and fungi (C, D) operational taxonomic units (OTUs) among four groups. US, UR, IS, and IR represent the uninfested rhizosphere soil, uninfected plant root, infested rhizosphere soil, and infected plant root, respectively. Chao 1 means the total number of OTUs estimated by infinite sampling, and a higher number indicates a higher richness; the Simpson index measures community evenness, and the index increases as diversity decreases.
